# Pharmacological targeting of the IL-17/neutrophil axis attenuates calcific deposits in rat models of calciphylaxis

**DOI:** 10.1172/JCI190369

**Published:** 2025-08-15

**Authors:** Bo Tao, Edward Z. Cao, James Hyun, Sivakumar Ramadoss, Juan F. Alvarez, Lianjiu Su, Qihao Sun, Zhihao Liu, Linlin Zhang, Alejandro Espinoza, Yiqian Gu, Feiyang Ma, Shen Li, Matteo Pellegrini, Arjun Deb

**Affiliations:** 1 Division of Cardiology, Department of Medicine, David Geffen School of Medicine,; 2 UCLA Cardiovascular Theme, David Geffen School of Medicine,; 3 Department of Molecular, Cell and Developmental Biology, College of Letters and Sciences,; 4 Eli and Edythe Broad Center of Regenerative Medicine and Stem Cell Research,; 5 Molecular Biology Institute;; 6 California Nanosystems Institute, and; 7 Department of Human Genetics, David Geffen School of Medicine, UCLA, Los Angeles, California, USA.; 8 Department of Cell and Developmental Biology, Northwestern University, Feinberg School of Medicine, Chicago, Illinois, USA.

**Keywords:** Cell biology, Dermatology, Bone disease, Neutrophils, Skin

## Abstract

Calciphylaxis is a rare but life-threatening disorder characterized by ectopic calcification affecting the subcutaneous tissues and blood vessels of the skin. Survival rates are less than a year after diagnosis, and yet despite the severity of the condition, the pathobiology of calciphylaxis is ill understood. Here, we created animal models of calciphylaxis that recapitulated many characteristics of the human phenotype. We demonstrate that cutaneous calcification is preceded by inflammatory cell infiltration. We show that increased local skin inflammation, regardless of the inciting cause, in the presence of hypercalcemia and hyperphosphatemia contributes to cutaneous ectopic calcification. Genetically modified rodents lacking immune activation of T and B cells or NK cells are resistant to developing cutaneous calcification. Consistent with this, administration of the immunosuppressive cyclophosphamide reduced calcific deposits, as did T cell suppression with cyclosporine. We demonstrate that IL-17 is upregulated in calcific skin and neutrophils are the predominant cell type expressing IL-17 and tissue-nonspecific alkaline phosphatase (TNAP) that are necessary for ectopic calcification. Targeting IL-17 with a monoclonal antibody or using a myeloperoxidase inhibitor to blunt neutrophil activation notably attenuated calcific deposits in vivo. Taken together, these observations provide fresh insight into the role of the immune system and the IL-17/neutrophil axis in mediating ectopic calcification in rodent models of calciphylaxis.

## Abstract 2

This paper creates unique rodent models of calciphylaxis to demonstrate the pivotal role of systemic inflammation in cutaneous ectopic calcification and pharmacologically targets the T cell-neutrophil axis to treat calciphylaxis.

## Introduction

Calcinosis cutis, or ectopic calcification of the skin and subcutaneous tissues, occurs in a variety of conditions such as autoimmune diseases of the skin that are associated with tissue damage and subsequent dystrophic calcification of injured tissues (1). Calciphylaxis is an unusual form of calcinosis cutis characterized by ectopic calcification of the blood vessels and subcutaneous tissues and typically observed in individuals with end-stage renal disease (ESRD) (2–4). High circulating calcium and phosphate, which are often elevated in individuals with ESRD, are considered risk factors for the development of calciphylaxis (5). Calciphylaxis occurs in 1%–2% of individuals with ESRD but is increasingly recognized to occur in individuals with normal renal function, particularly after organ transplantation (6–8). Calcific deposits in calciphylaxis are typically calcium hydroxyapatite (9) and develop gradually, but accelerated deposition of ectopic calcification in the skin and subcutaneous adipose fat can occur as well (10). The subcutaneous tissue ischemia associated with arteriolar calcification as well as calcific deposits in the extravascular interstitium poses enormous problems for maintaining tissue integrity and repair (11, 12). Patients typically suffer from painful lesions and nonhealing ulcers, and most individuals die from wound infection (13). The diagnosis of calciphylaxis is associated with substantial mortality with estimated survival of a few months to a year (14, 15). Despite the enormous mortality associated with calciphylaxis, its pathogenesis remains ill understood, and there are no effective treatments for this condition (2, 4).

Individuals with ESRD commonly develop extraskeletal ectopic calcification but usually do not develop calciphylaxis (16). The underlying biological reasons why the skin gets involved as a site of ectopic calcification in individuals with calciphylaxis are not clear. Risk factors for developing calciphylaxis include conditions and diseases that cause hypercalcemia and hyperphosphatemia, autoimmune disorders, and disorders or drugs that promote a thrombotic state (2, 11, 15, 17–19). Inflammation has been considered to play an important role in ectopic calcification of soft tissues (20–22), and human imaging studies have confirmed the presence of active inflammation in sites of ectopic vascular calcification (23). In animal models, a strong association between inflammatory burden and osteogenic activity in atherosclerotic plaque has been noted (24, 25). Moreover, in longitudinal clinical studies, increased inflammation at baseline was associated with a markedly higher degree of calcification of the aortic wall (26). Inhibition of osteogenic activity to attenuate ectopic calcification in animal models also reduced inflammation (27). Taken together, these observations in both animal models and human studies demonstrate a strong association between inflammation and calcification. Risk factors for calciphylaxis such as ESRD, autoimmune disease, and diabetes are associated with a chronic inflammatory state (28, 29), but little is known about the role of inflammation in directly contributing to ectopic calcification of the skin in calciphylaxis.

Rodent models of calciphylaxis were initially developed decades ago (30–33), where the animal was initially “sensitized” to develop hypercalcemia and hyperphosphatemia with administration of a vitamin D analog. Next, an “inducer” such as ferric chloride that causes vascular thrombosis and intense inflammation of tissues was injected into the subcutaneous tissue (31, 33). The animals developed robust calcification of the affected skin region. We have recreated these models of calciphylaxis and demonstrate robust deposition of ectopic calcification in subcutaneous tissues with histological features recapitulating phenotypic features of human calciphylaxis. Using a variety of genetically modified rodents that are incapable of mounting a robust immune response, or broad immunosuppressives such as cyclophosphamide or cyclosporine, we show the pivotal causal role of the immune system in mediating ectopic calcification in animal models of calciphylaxis. We demonstrate that IL-17 is notably upregulated in calcific skin lesions, and tissue-nonspecific alkaline phosphatase, an enzyme regulating final deposition of calcium minerals, is expressed by neutrophils. Targeting the IL-17/neutrophil axis with a monoclonal antibody against IL-17a or a myeloperoxidase inhibitor to blunt neutrophil activation notably rescued ectopic calcification. Taken together, our observations suggest that targeting the immune system and more specifically the IL-17/neutrophil axis may attenuate ectopic calcification in calciphylaxis.

## Results

### A rodent model associated with cutaneous calcification with hypercalcemia, hyperphosphatemia, and cutaneous inflammation.

**Figure 1:**
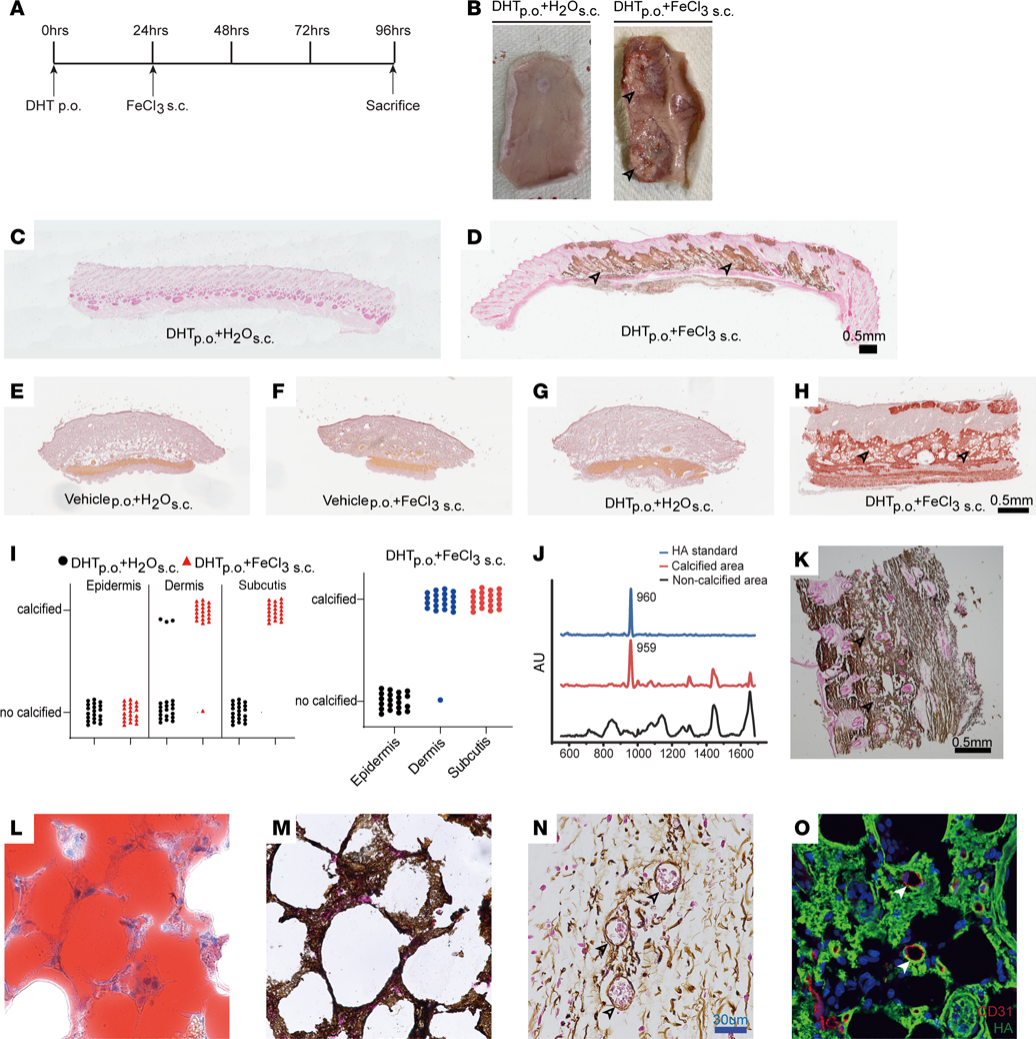
A rodent model of ectopic cutaneous calcification. (**A**) Sprague-Dawley rats were sensitized with dihydrotachysterol (DHT; 10 mg/kg, orally [p.o.]) and challenged with FeCl_3_ (25 μg/20 μL, s.c.) to induce ectopic tissue calcification in dorsal skin. (**B**) Gross anatomy of harvested dorsal skin. Black arrowheads indicate regions of ectopic mineralization in the dermal tissue. (**C** and **D**) Von Kossa staining of rat dermal tissue sensitized with DHT and injected with H_2_O (**C**) or FeCl_3_ (**D**). (Black arrowheads point to calcified regions. *n* = 4 animals per group.) Scale bar: 0.5 mm. (**E**–**H**) Alizarin red staining of rat dermal biopsies (4 mm). (**E**) Vehicle p.o. + H_2_O s.c. (**F**) Vehicle p.o. + FeCl_3_ s.c. (**G**) DHT p.o. + H_2_O s.c. (**H**) DHT p.o. + FeCl_3_ s.c. (Black arrowheads point to calcified regions. *n* = 4 animals per group.) Scale bar: 0.5 mm. (**I**) Quantitative analysis of site of von Kossa staining–positive regions in rat skin (DHT p.o. + FeCl_3_ s.c.: *n* = 20 animals, 20 sites; subcutis: 20/20; dermis: 19/20; epidermis: 0/20; DHT p.o. + H_2_O s.c.: *n* = 20 animals, 20 sites; subcutis: 0/20; dermis: 3/20; epidermis: 0/20). (**J** and **K**) Raman spectroscopy analysis of hydroxyapatite and calcified and non-calcified dermal tissue, with representative von Kossa–stained dermal tissue used for Raman microscopy. Scale bar: 0.5 mm. (**L** and **M**) Assessment of calcific deposits around adipose tissue in calcified dorsal skin using Oil Red O (adipocyte identification) and von Kossa staining (*n* = 3 animals). (**N** and **O**) Histology and immunofluorescent staining of CD31 in calcified regions of skin demonstrated calcific regions (green) in the periendothelial region (red, arrowheads) (representative images, *n* = 3 animals). Scale bar: 30 μm.

We recreated animal models of calciphylaxis, in which adult (4–6 weeks old) Sprague-Dawley rats were first given dihydrotachysterol (DHT) orally (Figure 1A). DHT is a vitamin D analog (34) and induces hypercalcemia and hyperphosphatemia, which are risk factors for calciphylaxis (2). Vitamin D is also used in other murine models of ectopic calcification, and initiation of vitamin D therapy has been associated with higher risk of calciphylaxis in humans (35, 36). Consistent with known effects of DHT, we observed increased serum calcium and phosphate in animals 24 hours following DHT administration compared with animals that received water (Supplemental Table 1; supplemental material available online with this article; https://doi.org/10.1172/JCI190369DS1). Twenty-four hours after administration of DHT, we injected the animals with ferric chloride subcutaneously.

FeCl_3_ is prothrombotic and proinflammatory, and vascular thrombosis and inflammation are thought to contribute to the pathogenesis of calciphylaxis (2). Within 3 days of FeCl_3_ injection, the subcutaneous tissue in the region of skin injected with FeCl_3_ demonstrated that hardening and calcific plaques were visible on gross inspection of the harvested skin (Figure 1B). Histological stains with von Kossa demonstrated extensive calcium deposits in the animals that received oral DHT but not in control groups that received subcutaneous injections of water instead of FeCl_3_ (Figure 1, C and D).

To confirm these observations, we also stained sections of the affected skin with alizarin red and observed extensive mineralized deposits in the skin of DHT+FeCl_3_–injected animals, in contrast to control groups that either did not receive DHT or were injected with water instead of FeCl_3_ after oral DHT administration (Figure 1, E–H). Analysis of histological sections to determine the site of calcified plaques demonstrated deposition of calcium in the dermis and subcutaneous tissue (Figure 1I). Next, to determine the chemical identity of the mineralized deposits, we subjected the calcified skin tissue to Raman microscopy and observed the deposits to be calcium hydroxyapatite, the predominant chemical deposited in human lesions of calciphylaxis as well (Figure 1, J and K).

Ectopic calcification in calciphylaxis is known to affect regions with abundant adipocytes. We performed Oil Red O staining to identify subcutaneous fat, and von Kossa staining to identify calcific deposits demonstrated the location of the calcific deposits to be around adipose tissue (Figure 1, L and M). Histological examination of blood vessels within the affected region demonstrated calcification of the walls of the blood vessels (Figure 1N), and double immunofluorescent staining with endothelial cells and calcium hydroxyapatite demonstrated the presence of mineralized deposits in the peri-endothelial region (Figure 1O).

Calciphylaxis is twice as common in females as in males (4), and hence female Sprague-Dawley rats were used. However, using a similar strategy of DHT and FeCl_3_ injection, we also observed a similar degree of ectopic cutaneous calcification in male rats, demonstrating that this model of calciphylaxis is not sex dependent (Supplemental Figure 1). Taken together, this model of cutaneous calcification recapitulates phenotypic features of human calciphylaxis with abundant extracellular and vascular calcification in the skin of affected animals.

### Inflammation precedes the development of calcification.

**Figure 2:**
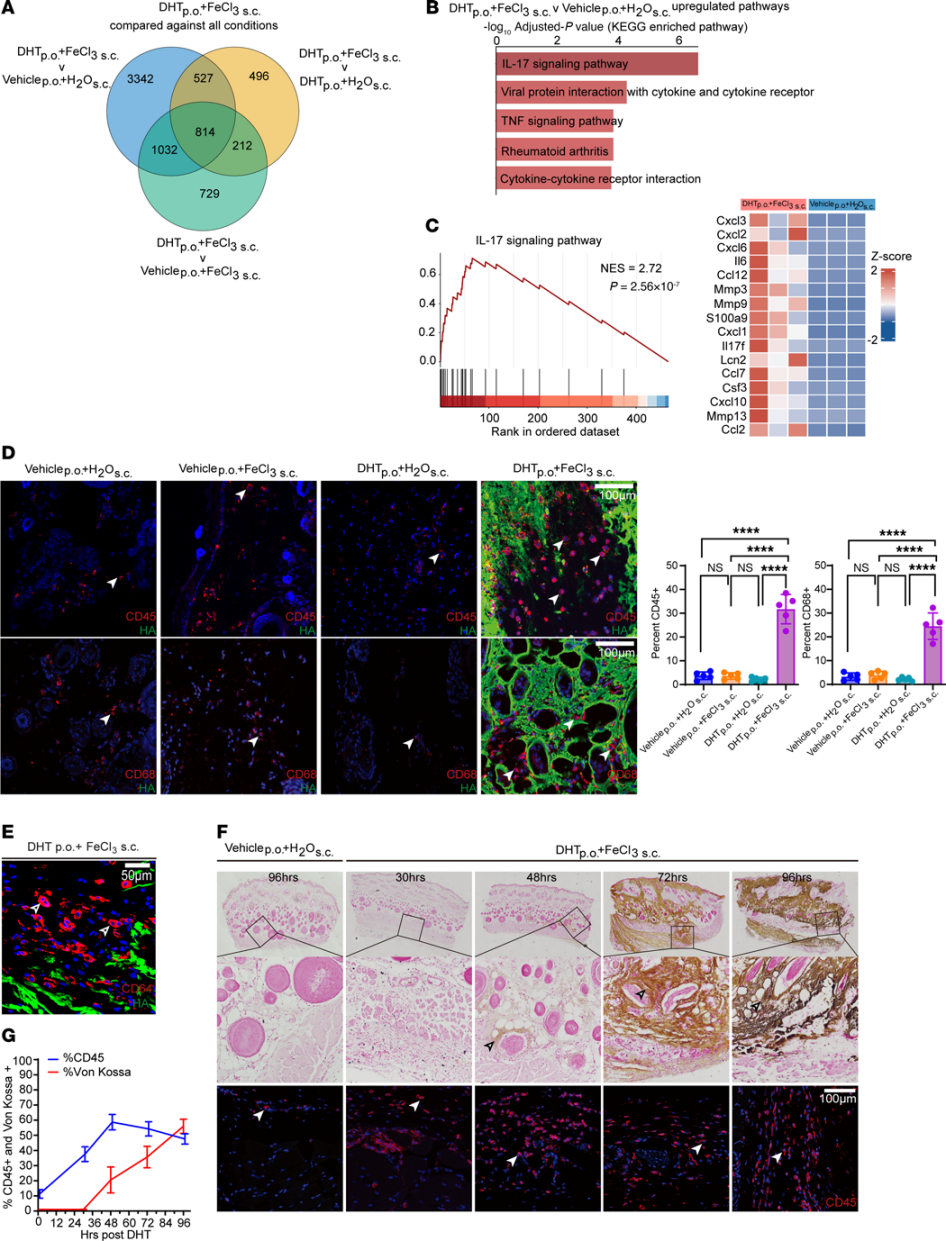
Inflammation is prominent and precedes calcific mineral deposits. (**A**) Venn diagram of RNA-Seq results from dermal tissues treated with vehicle p.o. + H_2_O s.c., DHT p.o. + H_2_O s.c., vehicle p.o. + FeCl_3_ s.c., and DHT p.o. + FeCl_3_ s.c. (*n* = 3 animals per group). (**B**) Top 5 Kyoto Encyclopedia of Genes and Genomes (KEGG) pathways associated with differentially expressed genes (DEGs) between DHT p.o. + FeCl_3_ s.c. and vehicle p.o. + H_2_O s.c. (*n* = 3 animals per group). (**C**) Enrichment score of the IL-17 signaling pathway and the top 16 leading-edge genes in DHT p.o. + FeCl_3_ s.c. versus vehicle p.o. + H_2_O s.c. analyzed by gene set enrichment analysis (GSEA). (**D**) Immunofluorescent staining of CD45 and CD68 in calcified skin regions reveals the presence of CD45 and CD68 (red, arrowheads) localized within the calcified areas (red, arrowheads, CD45/CD68; green, HA,hydroxyapatite) (representative images). Quantification of CD45^+^ cells and CD68^+^ cells revealed a robust increase in macrophage infiltration in the DHT+FeCl_3_ group (*n* = 5 animals per group). Scale bars: 100 μm. (**E**) Representative immunofluorescent staining image of CD64 in calcified skin reveals CD64^+^ cells (red, arrowheads) localized within hydroxyapatite-rich (HA-rich) calcified regions (green) (*n* = 4 animals). Scale bars: 50 μm. (**F**) Time-course histological and immunofluorescent staining of CD45 at 30, 48, 72, and 96 hours in DHT-treated rats injected with FeCl_3_ (*n* = 6 animals per group). Scale bars: 100 μm. (**G**) Temporal analysis of von Kossa–positive and CD45-positive cells in rat skin. Data are represented as mean ± SEM. ****P < 0.0001, 2-tailed Student’s *t* test.

To determine changes in tissue gene expression following ectopic calcification, we harvested the tissues at 4 days after oral DHT administration or 3 days after subcutaneous injection of FeCl_3_ and performed bulk tissue transcriptomics. As controls, we also harvested skin of animals identically treated with DHT but not given FeCl_3_ and another group that was injected subcutaneously with FeCl_3_ but did not receive DHT (Figure 2A). We selected genes differentially expressed in the skin of animals that exhibited ectopic calcification compared with the other groups. We observed that 814 genes were differentially expressed in the calcific group compared with the other control noncalcific groups (Figure 2A). Gene ontology analysis demonstrated differential expression of genes related to inflammation including IL-17 pathway and other inflammatory signaling pathways implicated in rheumatoid arthritis and TNF signaling pathways (Figure 2B). The genes related to the IL-17 pathway were the most extensively expressed (Figure 2C). IL-17 is known to be a master regulator controlling diverse aspects of immune regulation including induction of cytokines and transcription factors, and IL-17 activity has been found to be dysregulated in various autoimmune diseases of the skin (37). We observed that various chemokines known to be downstream of IL-17 were extensively upregulated in calcific tissues (Figure 2C). To confirm the presence of inflammatory cell recruitment in calcific tissues, we performed immunostaining and observed the presence of abundant CD45^+^ (hematopoietic cell marker) and CD68^+^ macrophages in calcified skin tissue compared with the other control groups (Figure 2D) and confirmed the presence of macrophages with the more specific macrophage marker CD64 (Figure 2E). To determine the temporal changes in recruitment of inflammatory cells following administration of DHT orally and FeCl_3_ subcutaneously, we harvested the skin tissue at 30, 48, 72, and 96 hours (time counted from DHT administration) and performed immunostaining for CD45^+^ and histological staining with von Kossa to detect calcium. We observed abundant accumulation of CD45-expressing inflammatory cells at 30 and 48 hours before the onset of observable calcium deposition (Figure 2F). These observations demonstrate that inflammatory cell recruitment precedes the onset of calcification by 24–48 hours (Figure 2G).

### Single-cell transcriptomics demonstrates distinct transcriptomic inflammatory signatures associated with ectopic calcification.

Our data demonstrate the accumulation of inflammatory cells prior to the onset of calcification (detected by histological methods) and the upregulation of inflammatory gene expression pathways. To obtain greater insight into the cellular transcriptome associated with such rapid and extensive tissue calcification, we performed single-cell transcriptomics. After DHT and FeCl_3_ administration, as described earlier, we harvested the skin at 3 days after FeCl_3_ injection, digested the skin tissue to isolate the cell population, and subjected the cells to single-cell RNA-Seq. Controls included animals that were injected with water and thus had noncalcified skin.

**Figure 3:**
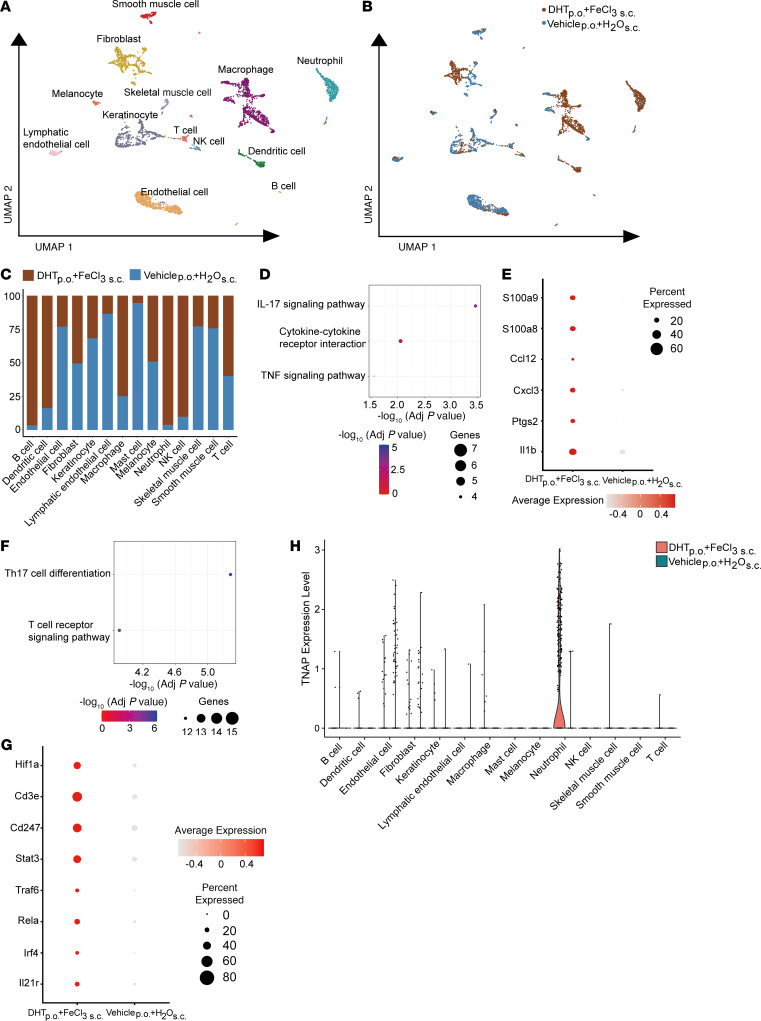
Single-cell RNA-Seq analysis of skin from calcific versus non-calcific tissue. (**A**) UMAP representation illustrating distinct cell cluster phenotypes within injured skin (*n* = 3 animals per group). (**B**) Distribution of cells from groups treated with DHT p.o. + FeCl_3_ s.c. and vehicle p.o. + H_2_O s.c. across these clusters. (**C**) Proportions of cell types in DHT p.o. + FeCl_3_ s.c. and vehicle p.o. + H_2_O s.c. (**D**) KEGG pathway analysis demonstrating the upregulation of inflammatory pathways in macrophages of DHT p.o. + FeCl_3_ s.c. versus vehicle p.o. + H_2_O s.c. (**E**) Dot plot highlighting DEGs of IL-17 signaling pathway between DHT p.o. + FeCl_3_ s.c. and vehicle p.o. + H_2_O s.c. (**F**) KEGG analysis demonstrating principal pathways upregulated in T cells of DHT p.o. + FeCl_3_ s.c. versus vehicle p.o. + H_2_O s.c. (**G**) Dot plot illustrating DEGs associated with the Th17 cell differentiation pathway in groups treated with DHT p.o. + FeCl_3_ s.c. and vehicle p.o. + H_2_O s.c. (**H**) Violin plot showing TNAP expression in specific cell populations in skin of DHT p.o. + FeCl_3_ s.c. and vehicle p.o. + H_2_O s.c.

Uniform manifold approximation and projection (UMAP) analysis demonstrated the typical cell population in murine skin, including keratinocytes, fibroblasts, endothelial cells, lymphatics, and inflammatory cells including macrophages, T cells, B cells, dendritic cells, and neutrophils (Figure 3A and Supplemental Figure 2). Distribution of the control and experimental phenotypes across these cell clusters showed robust increase in inflammatory cell types including neutrophils, macrophages, dendritic cells, and B, T, and NK cells (Figure 3, B and C). Fibroblasts and mesenchymal cells with osteogenic gene expression signatures have been thought to contribute to ectopic calcification (38–40), but we did not find any considerable differences in the fraction of fibroblasts/stromal cells between calcific and noncalcific skin, thus suggesting a more dystrophic form of ectopic calcification in this model.

Gene expression of osteogenic transcription factors such as Runx2 or Sox9 was not differentially expressed in calcific versus noncalcific skin (Supplemental Figure 3, A and B), and immunostaining confirmed the absence of Runx2 or Sox9 in calcific skin (Supplemental Figure 3C). Anti-calcific factors such as MGP also did not exhibit differential expression on immunostaining (Supplemental Figure 3D).

We next examined the gene expression of inflammatory cells between calcific and noncalcific skin and observed that the Il-17 signaling pathway was the most robustly differentially upregulated pathway (Figure 3D). Genes belonging to the IL-17 pathway such as CxCl3 and IL1b were robustly upregulated in inflammatory cells of calcific versus noncalcific skin (Figure 3E). The IL-17 pathway regulates the formation of Th17 cells, and examination of gene expression pathways differentially expressed in T cells demonstrated the Th17 pathway to be specifically upregulated in T cells; pathways related to T cell receptor expression and activation were upregulated as well (Figure 3F). Genes related to the Th17 pathway in T cells were robustly upregulated, including transcriptional regulators such as Stat3, Irf4, and Rela, as well as Th17-associated markers like IL21r and Hif1a (Figure 3G).

As tissue alkaline phosphatase is thought to play a critical role in ectopic calcification (41), we next examined the cell population in the calcific tissue that expressed tissue-nonspecific alkaline phosphatase (TNAP) and observed that TNAP was predominantly if not exclusively expressed by neutrophils in the calcific region (Figure 3H). Taken together, these findings suggest a robust inflammatory transcriptomic response and TNAP expression in neutrophils in the calcified region.

### Injection of other agents that induce sterile inflammation also leads to ectopic calcification.

**Figure 4:**
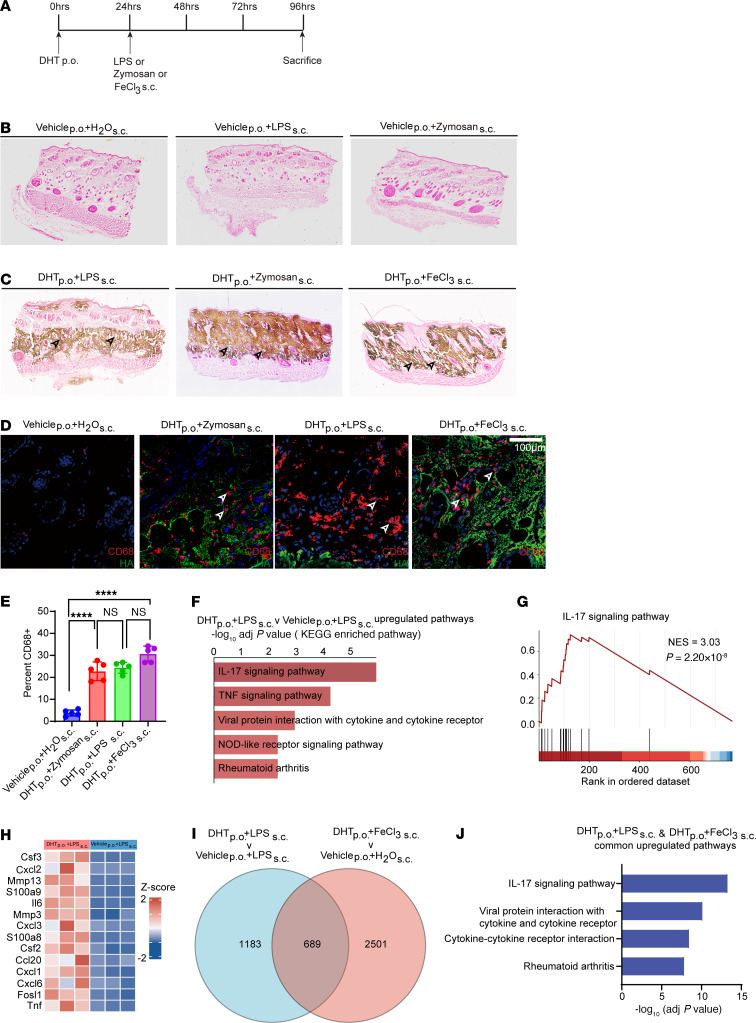
Administration of sterile inflammation–inducing agents after DHT administration also leads to ectopic calcification. (**A**) Experimental scheme of LPS, FeCl_3_, and zymosan treatment. (**B** and **C**) Von Kossa staining of rat dermal tissues to identify calcific regions in controls (**B**) and animals that received DHT (**C**) (arrowheads; representative images; *n* = 3 animals per group). (**D** and **E**) Immunofluorescent staining of CD68 in calcified skin regions (red, arrowheads, CD68; green, HA, hydroxyapatite). (**D**) and quantification of inflammatory infiltrate (**E**) (representative images; *n* = 5 animals per group). Scale bar: 100 μm. (**F**) Top 5 KEGG pathways associated with DEGs between DHT p.o. + LPS s.c. (*n* = 5 animals) and vehicle p.o. + LPS s.c. (*n* = 3 animals). (**G** and **H**) GSEA analysis of the IL-17 signaling pathway showing enrichment scores (**G**) and the top 14 leading-edge genes (**H**) in DHT p.o. + LPS s.c. versus vehicle p.o. + LPS s.c. (**I**) Venn diagram depicting common DEGs between DHT p.o. + LPS s.c. (*n* = 5 animals) and vehicle p.o. + LPS s.c. (*n* = 3 animals) and between DHT p.o. + FeCl_3_ s.c. (*n* = 4 animals) and vehicle p.o. + H_2_O s.c. (*n* = 3 animals). (**J**) Shared upregulated pathways between DHT p.o. + LPS s.c. and vehicle p.o. + LPS s.c. and between DHT p.o. + FeCl_3_ s.c. and vehicle p.o. + H_2_O s.c. are identified and highlighted. Data are represented as mean ± SEM. ****P < 0.0001, 2-tailed Student’s *t* test.

We next investigated whether the presence of cutaneous inflammation was sufficient to induce ectopic calcification following administration of DHT. We thus injected a group of animals with either lipopolysaccharide (LPS) or zymosan, two chemicals that have been extensively used to induce sterile inflammation (Figure 4A). We observed that subcutaneous injection of either LPS or zymosan induced ectopic cutaneous calcification and the gross appearance and histologic distribution were comparable to those induced by FeCl_3_. (Figure 4, B and C). In the absence of prior oral DHT, LPS or zymosan injections did not cause ectopic calcification, demonstrating the critical necessity of hypercalcemia and hyperphosphatemia (Figure 4B). Abundant numbers of inflammatory cells were observed in the affected skin tissue of animals injected with zymosan or LPS, and the degree of inflammation was similar across the FeCl_3_, zymosan, and LPS groups (Figure 4, D and E). We next performed bulk tissue transcriptomics and examined pathways differentially expressed in the DHT+LPS (calcific) versus LPS-alone groups. We observed that pathways of inflammation including IL-17 and TNF-α signaling pathways were differentially expressed in tissue calcified with DHT+LPS (Figure 4F) and were similar to pathways differentially upregulated with DHT+FeCl_3_. The IL-17 pathway was again the most dramatically upregulated in animals receiving DHT and LPS (Figure 4G). Calcific tissues of animals that received DHT+LPS demonstrated that similar chemokines were upregulated in comparison with calcific tissues of animals that received DHT+FeCl_3_ (Figure 4H). To find common sets of genes that are upregulated in calcific skin tissue regardless of the nature of the inflammatory stimulus, we compared the differentially expressed genes in calcific tissues of animals that received DHT+LPS or DHT+FeCl_3_ versus their respective uncalcified-skin controls. We observed that 689 genes were differentially expressed in both groups (Figure 4I), and Gene Ontology analysis demonstrated that IL-17 signaling and cytokine/chemokine pathways were commonly upregulated in both groups of animals (LPS and FeCl_3_) exhibiting ectopic skin calcification (Figure 4J).

### Administration of the drug clodronate that ablates macrophages prevents ectopic calcification.

We next investigated whether suppression of the immune response would attenuate ectopic calcification. The drug clodronate has been extensively used to ablate monocyte-derived macrophages. We administered clodronate intraperitoneally (i.p.) 72 hours before administration of DHT and administered a second dose on the day of DHT administration (Supplemental Figure 4A). FeCl_3_ was injected 24 hours later, and the animals were harvested at 4 days to determine the effects of clodronate on ectopic calcification (Supplemental Figure 4A). We observed that the skin of animals that received clodronate remained soft and viable and there were no calcific lesions on gross examination, in contrast to vehicle-injected controls, which exhibited robust calcification (Supplemental Figure 4B). Histological staining with von Kossa identified extensive calcific deposits in the DHT+FeCl_3_ group that received vehicle, but in the DHT+FeCl_3_ group that received clodronate, there was a complete absence of calcification (Supplemental Figure 4C). Immunostaining for CD45^+^ hematopoietic cells and CD68-expressing macrophages demonstrated an extensive reduction in the skin of animals that received clodronate (Supplemental Figure 4, D and E). To independently quantitate the effects of clodronate on macrophage ablation, we examined splenic macrophages by immunostaining for CD68 expression in the spleen and observed an extensive reduction in the numbers of splenic macrophages, confirming the known effect of clodronate in ablating macrophages (Supplemental Figure 4, F and G). We next examined the number of circulating CD11b^+^ cells (monocyte lineage) in circulating blood, and consistent with the known pro-apoptotic effects of clodronate on the monocyte/macrophage lineage, we observed a robust decrease in CD11b^+^ circulating monocyte counts (Supplemental Figure 4H). Examination of peripheral blood white blood cell and neutrophil counts did not demonstrate any significant differences in animals that received clodronate (Supplemental Table 2). As recent data suggest that clodronate could exert effects on neutrophil function (42), we performed immunostaining for myeloperoxidase (MPO; abundant in neutrophils) and observed a striking reduction in MPO staining in animals that received clodronate (Supplemental Figure 4, I and J). Serum biochemistry did not demonstrate any significant differences between vehicle- and clodronate-treated animals except a mild decrease in serum phosphate (Supplemental Table 3). Taken together, these observations suggest that clodronate decreases circulating monocytes and macrophages in tissue, exerts effects on neutrophils in tissue, and leads to complete rescue of ectopic calcification.

### Genetically modified rodents with deficiencies in B, T, and NK cell development do not develop ectopic calcification.

**Figure 5:**
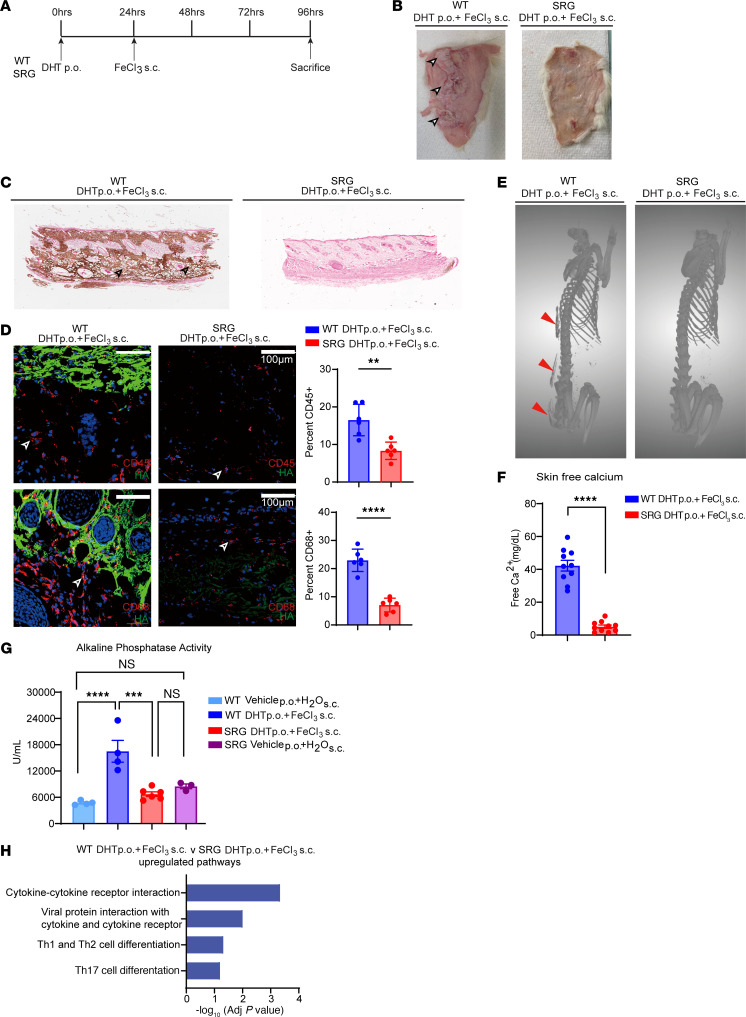
Genetic deletion of B, T, and NK cells (SRG rats) inhibits the formation of ectopic calcification. (**A**) Experimental scheme comparing the ability of WT and SRG rats treated with DHT p.o. + FeCl_3_ s.c. to exhibit ectopic cutaneous calcification. (**B**) Gross images of dermal tissue in WT and SRG rats. Black arrowheads point to calcific regions. (**C**) Representative von Kossa–stained images highlighting calcified regions in rat dermal tissue. (**D**) Immunofluorescent staining of CD45^+^ and CD68^+^ cells in WT and SRG rat dermal tissue (representative images; *n* = 6 animals per group). Scale bars: 100 μm. (**E**) CT scan of rat dermal tissue in WT and SRG rats (arrowheads point to extraskeletal calcification) (WT, *n* = 4 animals; SRG, *n* = 6 animals). (**F**) Quantification of free calcium levels in rat skin dermal tissues (*n* = 10 animals per group). (**G**) Assessment of alkaline phosphatase activity in rat dermal tissues (TNAP, tissue-nonspecific alkaline phosphatase) (WT DHT p.o. + FeCl_3_ s.c.: *n* = 4 animals; WT vehicle p.o. + H_2_O s.c.: *n* = 4 animals; SRG DHT p.o. + FeCl_3_ s.c.: *n* = 6 animals; SRG vehicle p.o. + H_2_O s.c.: *n* = 3 animals). (**H**) Upregulated pathways identified in WT versus SRG rats after DHT p.o. + FeCl_3_ s.c. (*n* = 4 animals per group). Data are represented as mean ± SEM. **P < 0.01, ***P < 0.001, ****P < 0.0001, 2-tailed Student’s *t* test.

As clodronate is a bisphosphonate and could potentially exert independent inhibitory effects on mineralization, we adopted a genetic approach to determine the effects of the immune system on ectopic calcification. The SRG rat (Sprague-Dawley genetic background) is an inbred rat that is immunodeficient secondarily to knockout mutations in key immune development genes such as Rag2 and Il2Rγ; it demonstrates absence of T, B, and NK cells (43). We subjected the SRG rat to ectopic calcification of the skin by administering DHT orally and then injecting FeCl_3_ subcutaneously (Figure 5A). Wild-type (WT) Sprague-Dawley rats were used as controls and developed calcific plaques in the skin at the site of FeCl_3_ administration, but there was complete absence of hardening of the skin in SRG rats on gross examination (Figure 5B). Histological staining with von Kossa demonstrated a complete absence of calcification in the SRG rats compared with WT control animals (Figure 5C). Circulating white blood cells as well as neutrophils were markedly decreased in SRG rats compared with WT animals (Supplemental Figure 5, A and B). Immunostaining for hydroxyapatite and CD45^+^ and CD68^+^ cells demonstrated robust reduction in hematopoietic cells and macrophages with complete absence of hydroxyapatite deposition (Figure 5D). Immunostaining for neutrophils (MPO marker) or T cells (Cd3^+^) demonstrated robust reduction in skin of SRG rats consistent with the aberrant immune system in these animals (Supplemental Figure 5, C and D). We subjected the SRG and WT animals to CT scanning and observed extraskeletal subcutaneous calcification in WT rats but complete absence of ectopic calcification in the SRG animals (Figure 5E). Measurement of calcium content in the skin demonstrated dramatic reduction of calcium mineral in the SRG rats consistent with decreased hydroxyapatite formation (Figure 5F). Serum biochemistry did not show any significant differences between SRG and WT animals; mild depression of serum calcium was noted in SRG animals (Supplemental Table 4). TNAP activity is critical for bone formation and ectopic mineralization; TNAP is the enzyme that liberates free phosphate for hydroxyapatite formation and growth (41). We measured tissue alkaline phosphatase activity in the skin and observed that in lesions of ectopic calcification, following DHT+FeCl_3_ administration, there was dramatic increase in alkaline phosphatase activity, but this rise was completely blunted in the skin of SRG animals (Figure 5G). The alkaline phosphatase level was not markedly higher in the SRG animals treated with DHT and FeCl_3_ than in the SRG animals treated with water (Figure 5G). Next, we performed bulk transcriptomics and observed that SRG animals compared with WT animals exhibited downregulation of cytokines, T cell differentiation, and particularly Th17 cell differentiation, which is regulated by the IL-17 pathway (Figure 5H). In addition, several chemokine ligands and chemokine receptors, such as Ccl1 and Ccl24, were sharply downregulated in the SRG rats along with downregulation of chemokine receptors and TNF pathways (Figure 5H and Table 1).

To confirm the phenotype observed in the SRG rats, we used another genetically modified immunodeficient rodent model. We used Rag2-KO rats, which are deficient in T and B cells but, unlike the SRG rats, not deficient in NK cells (43). We used a similar strategy of oral DHT administration and subcutaneous FeCl_3_ injection (Supplemental Figure 6A). The WT animals demonstrated robust calcification, but the Rag2-KO rats did not exhibit any ectopic calcification (Supplemental Figure 6B). These observations are consistent with the observations made in the SRG rat and suggest a critical role of the immune system in mediating ectopic calcification.

### Cyclophosphamide or cyclosporine prevents ectopic calcification of the skin.

**Figure 6:**
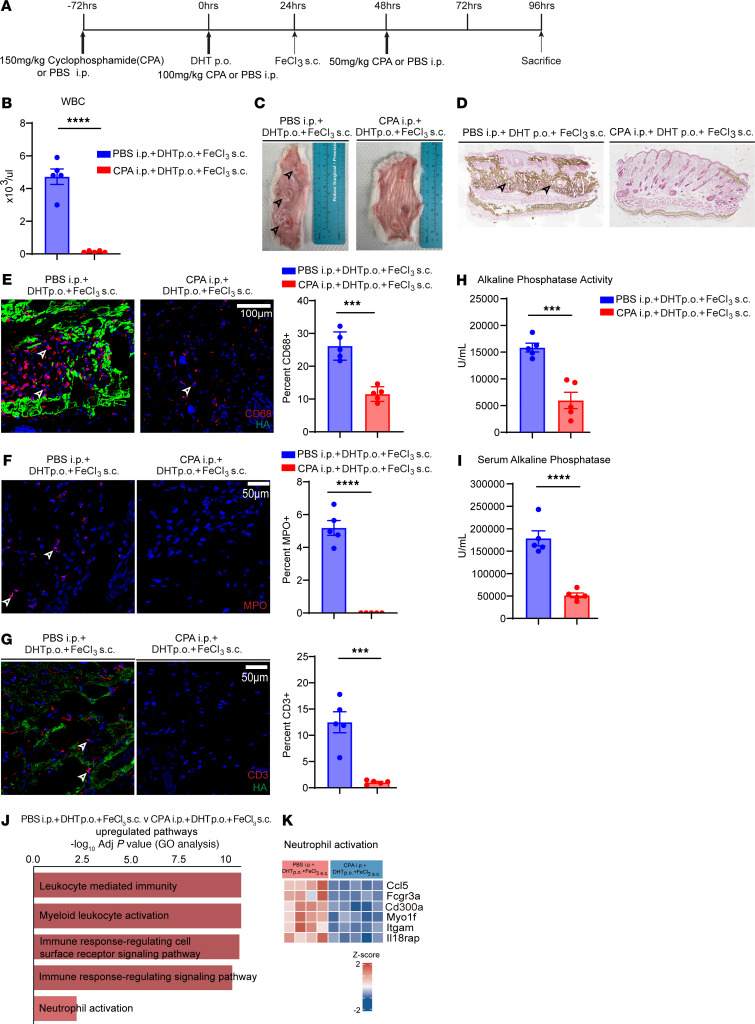
Cyclophosphamide-induced leukopenia rescues ectopic calcification. (**A**) Experimental scheme for neutrophil depletion using cyclophosphamide (CPA) in WT rats treated with DHT p.o. + FeCl_3_ s.c. (**B**) Comparison of WBC count in PBS- and CPA-treated rats after DHT p.o. + FeCl_3_ s.c. administration (*n* = 5 animals per group). (**C**) Gross images of rat dermal tissue in PBS- and CPA-treated rats. Black arrowheads point to calcific regions. (**D**) Von Kossa–stained rat dermal tissue highlighting calcification in PBS- and CPA-treated rats given DHT p.o. + FeCl_3_ s.c. (representative images; arrowheads point to calcific regions; *n* = 4 animals per group). (**E**) Immunofluorescent staining of CD68^+^ cells in PBS- and CPA-treated rats and quantification (representative images; *n* = 4 animals per group). Scale bar: 100 μm. (**F**) Immunofluorescent staining of myeloperoxidase (MPO) to identify neutrophils in PBS- and CPA-treated rats and quantification (representative images; *n* = 4 animals per group). Scale bar: 50 μm. (**G**) Immunofluorescence staining and quantification of CD3^+^ T cells in PBS- versus CPA-treated rats (*n* = 5 animals per group). Scale bar: 50 μm. (**H**) Alkaline phosphatase activity in rat dermal tissues in PBS- versus CPA-treated animals (*n* = 5 animals per group). (**I**) Serum alkaline phosphatase activity in rats treated with PBS or CPA (*n* = 5 animals per group). (**J**) Gene Ontology analysis associated with upregulated DEGs in PBS-treated (*n* = 4 animals) and CPA-treated rats following DHT p.o. + FeCl_3_ s.c. (*n* = 5 animals). (**K**) Enriched genes in immune response–regulating cell surface receptor signaling pathway. Data are represented as mean ± SEM. ***P < 0.001, ****P < 0.0001, 2-tailed Student’s *t* test.

Single-cell transcriptomics data have demonstrated markedly increased numbers of inflammatory cells including T cells, B cells, dendritic cells, neutrophils, and macrophages in the ectopic calcific lesions compared with noncalcified skin. Immunomodulatory signaling pathways such as the IL-17 pathway that play pivotal roles in T cell activation and autoimmune diseases of the skin were consistently found to be upregulated in skin of animals that exhibited calcification versus noncalcified skin. Neutrophils are known to be recruited to the inflammatory region by IL-17–mediated signaling, and neutrophils were the principal cells that expressed TNAP, an enzyme whose activity is known to be critical for tissue mineralization. We next investigated whether the widely used alkylating chemotherapeutic agent cyclophosphamide, which induces profound leukopenia and immunosuppression, would prevent ectopic calcification in the rodent model of calciphylaxis. We administered the drug cyclophosphamide (150 mg/kg i.p.) 3 days before DHT administration (100 mg/kg i.p.) and on the day of DHT administration; a final dose of 50 mg/kg i.p. was administered 24 hours after FeCl_3_ injection; and animals were harvested at 3 days after subcutaneous FeCl_3_ administration (Figure 6A). Cyclophosphamide-injected animals were profoundly leukopenic, consistent with the known effect of cyclophosphamide in inducing leukopenia (Figure 6B). Gross anatomic inspection demonstrated the absence of calcific plaques in cyclophosphamide-injected animals compared with vehicle-injected controls (Figure 6C). Histological analysis with von Kossa staining demonstrated complete absence of calcific deposits in the cyclophosphamide-injected animals (Figure 6D), and immunofluorescent staining demonstrated striking reduction in inflammatory macrophages in the region of skin injected with FeCl_3_ (Figure 6E). Serum biochemistry did not demonstrate any significant differences between cyclophosphamide- and vehicle-treated animals (Supplemental Table 5). Immunostaining with MPO to identify neutrophils demonstrated marked reduction of neutrophils in the skin of animals that received cyclophosphamide (Figure 6F), and T cell counts were also greatly decreased in skin of animals that received cyclophosphamide (Figure 6G). Tissue and serum alkaline phosphatase activity was considerably decreased in the skin of animals that received cyclophosphamide (Figure 6, H and I). Gene expression analysis of skin of animals injected with cyclophosphamide versus vehicle-injected controls demonstrated suppression of leukocyte-related immunity and leukocyte activation in the cyclophosphamide-injected animals (Figure 6, J and K). We also investigated whether cyclophosphamide administered after the development of calcific deposits could reverse the calcific phenotype. For this purpose, we administered cyclophosphamide 48 hours after FeCl_3_ injection when deposition of calcium has already been initiated (Supplemental Figure 7A). In comparison with vehicle-injected controls, we did not observe reduction of calcific deposits by cyclophosphamide when administered after the onset of calcification, even though peripheral blood leukopenia was confirmed (Supplemental Figure 7, B and C). Taken together, these observations suggest that immunosuppression prevents but does not reverse ectopic cutaneous calcification.

To corroborate the role of immunosuppressives in treating ectopic calcification, we next tested the effects of the drug cyclosporine. Cyclosporine is a widely used immunosuppressive that inhibits the calcineurin pathway to inhibit T cell activation and is also known to inhibit the IL-17 pathway. We administered cyclosporine to the animals starting 7 days before DHT administration and continuing until 11 days (Supplemental Figure 8A). We observed that cyclosporine completely rescued the development of calcific plaques in the skin (Supplemental Figure 8B), and histological examination with von Kossa staining demonstrated complete absence of calcific deposits (Supplemental Figure 8C). Immunofluorescent staining demonstrated dramatically decreased inflammatory infiltrate (Supplemental Figure 8, D and E), and this was associated with decreased TNAP expression (Supplemental Figure 8F).

### IL-17 blockade with a monoclonal antibody dramatically attenuates ectopic calcification.

Transcriptomic analysis of tissues affected by calcification using a variety of inflammatory agents, including agents that lead to sterile inflammation, has demonstrated upregulation of IL-17 pathways. IL-17 has been implicated in a variety of autoimmune skin diseases, such as psoriasis and eczema. We next investigated whether specific targeting of IL-17 could rescue calcification in the rodent model of calciphylaxis.

**Figure 7:**
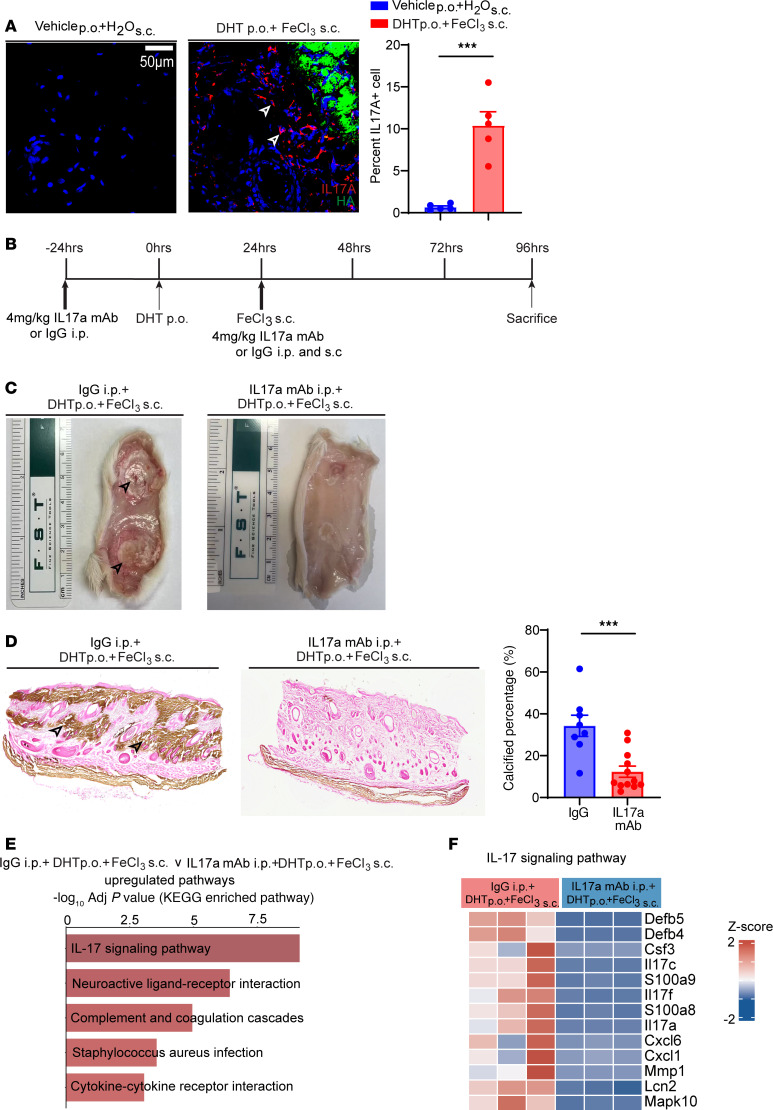
IL-17a blockade prevents skin calcification and inflammation in the DHT+FeCl_3_–induced rat model. (**A**) Immunofluorescence staining and quantification of IL-17a^+^ cells in skin tissue from rats treated with DHT p.o. + FeCl_3_ s.c. or vehicle p.o. + H_2_O s.c. (representative images; *n* = 5 animals per group). Scale bar: 50 μm. (**B**) Schematic of the IL-17a mAb experimental timeline. (**C**) Gross images of dorsal skin lesions. Black arrowheads show visible regions of calcification. (**D**) Representative von Kossa–stained images of rat dermal tissue highlighting calcified regions (arrowheads). Quantification of calcified percentage in the IL-17a mAb–treated group (*n* = 12) compared with IgG control (*n* = 8). (**E**) Top 5 KEGG pathways enriched in DEGs between IgG-treated (*n* = 3 animals) and IL-17a mAb–treated rats following DHT p.o. + FeCl_3_ s.c. (*n* = 3 animals). (**F**) Enriched genes in IL-17 signaling pathway. Data are represented as mean ± SEM. ***P < 0.001, 2-tailed Student’s *t* test.

We performed immunostaining for IL-17a and observed noticeable upregulation of IL-17a in calcific tissues, particularly surrounding the calcific edges of tissue (Figure 7A). To determine the specific role of IL-17 in mediating calcific deposits, we injected the animals with a monoclonal antibody targeting IL-17a (IL-17a mAb, Bio X Cell, BP0173). The mAb or control IgG was administered i.p. 24 hours before DHT administration followed by a repeat dose both i.p. and subcutaneously 24 hours after DHT administration (Figure 7B). Animals were given DHT and FeCl_3_ to induce calcification, and we observed that at harvest, skin of animals that received IL-17 in contrast to IgG did not show any evidence of calcific plaques on gross inspection (Figure 7C). Von Kossa staining demonstrated near-complete rescue of calcific deposits by IL-17mAb (Figure 7D). Serum biochemistry demonstrated noticeable decrease in blood urea nitrogen and creatinine in the IL-17–treated animals suggestive of physiological benefit to kidney function in this model (Supplemental Table 6). We next performed bulk tissue transcriptomics and observed that gene expression of IL-17 pathways was considerably downregulated in animals that received IL-17mAb compared with control IgG (Figure 7E), and specific genes of the IL-17 pathway, including many chemokines and members of the IL-17 family, were considerably downregulated in the skin tissue of animals treated with IL-17mAb (Figure 7F), confirming effective inhibition of the pathway. Taken together, these observations suggest that pharmacological antagonism of the IL-17 pathway can prevent ectopic cutaneous calcification.

### Administration of MPO inhibitor to blunt neutrophil-mediated inflammation also rescues calcification.

There is increasing evidence to suggest that neutrophils in inflamed tissue can be a source of IL-17 (44).We performed immunostaining of calcified tissue and observed that neutrophils identified by MPO expression also expressed IL-17a (Supplemental Figure 9A). As drugs such as cyclophosphamide that cause leukopenia prevented calcification, we next investigated whether administration of an MPO inhibitor to blunt neutrophil-mediated response could also attenuate ectopic cutaneous calcification.

For this purpose, we administered the MPO inhibitor 4-aminobenzohydrazide 24 hours before the administration of oral DHT and continued it daily until the animals were sacrificed (Supplemental Figure 9B). DHT and FeCl_3_ were administered to induce calcification (Supplemental Figure 9B). We observed that at harvest, on gross inspection, the skin of animals that received the MPO inhibitor did not show any evidence of calcified plaques (Supplemental Figure 9C), and von Kossa staining demonstrated marked attenuation of ectopic calcification (Supplemental Figure 9D). Serum biochemistry did not show any significant differences between the MPO inhibitor– and vehicle-treated animals (Supplemental Table 7). Taken together, these observations suggest that antagonism of the IL-17/neutrophil activation axis rescues ectopic calcification in calciphylaxis.

## Discussion

Rodent models of calciphylaxis were described more than five decades ago where animals injected with a vitamin D analog to induce hypercalcemia and hyperphosphatemia developed rapid ectopic calcification of skin at the site of injection of an irritant stimulus (31, 33). This model shares commonalities with the disease phenotype of human calciphylaxis with similar risk factors, such as vitamin D and presence of hypercalcemia or hyperphosphatemia, that are known to trigger calciphylaxis in humans and the deposition of calcium around adipose tissue that is characteristically seen in human calciphylaxis. However, rodent models of calciphylaxis also harbor differences from human calciphylaxis. Arterial thrombosis is thought to be a key event in human calciphylaxis, but in the rodent model perivascular and endothelial calcification was predominant rather than thrombosis. Despite these differences, the phenotype of extravascular ectopic calcification as observed in human calciphylaxis was robustly observed in our model and enables the molecular investigation of potential therapies.

Ectopic calcification is thought to be an active process, cell mediated and analogous to bone formation rather than a passive precipitation of calcium and phosphate to form calcium hydroxyapatite (21). The rapidity of calcification seen in our model would argue against the generation or differentiation of mesenchymal or stromal cells into osteogenic cells, and gene expression of calcific tissue did not show any upregulation of an osteogenic gene expression program or decreased expression of anti-calcific genes such as matrix Gla protein (*MGP*). In contrast, the model of rapid ectopic calcification in this model is consistent with dystrophic calcification, a term used to describe rapid crystallization of calcium salts in tissue particularly after cell death or injury (45).

Little is known about the molecular regulation of dystrophic calcification, but our observations taken together strongly suggest that the immune system and in particular the IL-17 pathway play a pivotal role in ectopic calcification. On unbiased gene ontogeny analysis of calcific versus noncalcific skin, the IL-17 signaling pathway was found to be robustly upregulated in calcific lesions using different inducers. IL-17 has been described as inducing an osteogenic gene expression program in primary osteoblasts (46), including expression of alkaline phosphatase, and IL-17 has been shown to induce vascular mineralization of the aorta in ex vivo culture models (47). IL-17 has also been shown to be present in inflammatory conditions such as Kawasaki disease that are associated with aneurysm and subsequent vascular calcification, and IL-17 induced a calcific phenotype in human coronary artery smooth muscle cells when cultured with serum from individuals with Kawasaki disease (48). IL-17 has a complex interaction with neutrophils and is known to act on epithelial and stromal cells to secrete various chemokines that lead to neutrophil recruitment. However, neutrophils have also been observed to be a source of IL-17. Our observations demonstrate a causal role of IL-17 in mediating calcification in calciphylaxis, with administration of a monoclonal antibody targeting IL-17a substantially attenuating ectopic calcification deposition. TNAP is critically required for tissue calcification as it releases free phosphate ions, and neutrophils were observed to robustly express TNAP. Although neutrophils are not known to establish TNAP, neutrophil extracellular traps have been implicated in ectopic calcification serving as a nidus for calcium hydroxyapatite mineralization and growth. IL-17 is known to stimulate mesenchymal cell proliferation as well as differentiation and expression of TNAP to adopt an osteogenic phenotype (49). Inhibitors of neutrophil MPO also rescued ectopic calcification in the rodent model of calciphylaxis, and taken together our data would suggest that IL-17 likely leads to neutrophil activation and expression of TNAP.

IL-17 monoclonal antibodies that block IL-17a (e.g., secukinumab) are currently in clinical practice to treat autoimmune conditions such as psoriasis and psoriatic arthritis. Our data suggest that IL-17 antagonism may have a new role in the treatment of cutaneous ectopic calcification in the rare but life-threatening disease calciphylaxis.

## Methods

### Sex as a biological variable.

Our protocol was adapted from previously published studies that used only female rats for observations. This is scientifically justified as women have a 2-fold greater risk of developing calciphylaxis than men. Therefore, our study also focused exclusively on female rats. However, we have observed that this model also induces calcification in male rats to the same degree.

### Statistics.

All data are presented as mean ± SEM, and the value of *n* represents biological replicates. Statistical analysis was performed with GraphPad Prism 8.3 software, using Student’s *t* test (2-tailed), 1-way ANOVA with Tukey’s multiple-comparison test, or 2-way ANOVA with Šidák’s multiple-comparison test as appropriate. *P* less than 0.05 was considered statistically significant.

### Study approval.

All animal studies were approved by the Animal Research Committee of UCLA, and all animals were maintained at the UCLA vivarium in accordance with the policies laid out by the Association for Assessment and Accreditation of Laboratory Animal Care International.

### Data availability.

All RNA-Seq and single-cell RNA-Seq data are available in the NCBI’s Gene Expression Omnibus database (GEO GSE301731). Values for all data points in graphs are reported in the Supporting Data Values file.

Detailed methods are provided in Supplemental Methods.

## Author contributions

BT and EZC performed the majority of the experiments. AD conceptualized the project, designed experiments, supervised data collection and analysis, and wrote the manuscript. JH and JFA helped to harvest skin and serum. LS, QS, and LZ helped to cut slices and stain for histology. ZL performed assays and designed the mechanism Figure. SR performed bench experiments related to molecular assays of calcium and prepared RNA-Seq samples. AE, YG, FM, and MP analyzed single-cell and bulk RNA-Seq data. SL helped to modify figure formatting and do CT scanning.

## Supplementary Material

Supplemental data

Supporting data values

## Figures and Tables

**Table 1:**
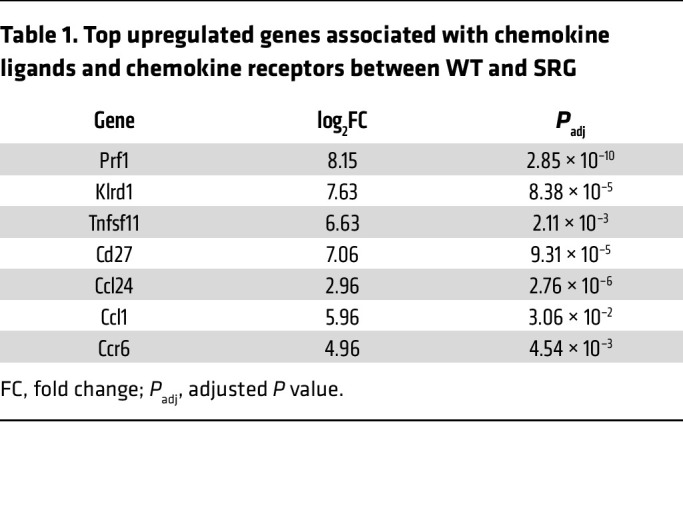
Top upregulated genes associated with chemokine ligands and chemokine receptors between WT and SRG

## Version 1 08/15/2025

In-Press Preview

## Version 2 10/01/2025

Electronic publication
