# Supplementary material for: Pharmacological targeting of the IL-17/neutrophil axis attenuates calcific deposits in rat models of calciphylaxis
Source: J Clin Invest. 2025 Aug 15;135(19):e190369. doi: 10.1172/JCI190369 (PMC12483568; doi:10.1172/JCI190369)
Supplement: Supplemental data [file jci-135-190369-s177.pdf]

Supplemental Materials for

Pharmacological targeting of the IL17/neutrophil axis attenuates calcific deposits in rat models of calciphylaxis

Bo Tao *[1,2,3,4,5,6] , Edward Cao *[1,2,3,4,5,6], James Hyun[1,2,3,4,5,6], Sivakumar Ramadoss [1,2,3,4,5,6], Juan Felipe Alvarez[1,2,3,4,5,6], Lianjiu Su[1,2,3,4,5,6], Qihao Sun[1,2,3,4,5,6], Zhihao Liu[1,2,3,4,5,6], Linlin Zhang[1,2,3,4,5,6], Alejandro Espinoza[7], Yiqian Gu[3,4,5,6], Feiyang Ma[8], Shen Li[1,2,3,4,5,6], Matteo Pellegrini[3,4,5,6], Arjun Deb**[1,2,3,4,5,6]

Correspondence to: adeb@mednet.ucla.edu

The following is included in the supplementary material.

Supplemental Methods

Figure S1 to S9

Table S1 to S7

References

Supplemental Methods

Rat models of calciphylaxis

Female Sprague-Dawley rats (~130g, Charles River, 400-SAS-SD) were sensitized with dihydrotachysterol (DHT; Sigma, 1204000) administered via oral gavage (Kent Scientific, FNC1632) at a dose of 10mg/kg in corn oil (Fisher Scientific, S25271) [1,2]. After 24 hours, rats were challenged with a subcutaneous delivery of 25μg/200ul $FeCl_3$ (Sigma, 157740) in H_2O along the dorsal midline. Tissues were collected 96 hours after oral gavage of DHT.

To assess the effects of macrophage depletion on the development of ectopic calcification, rats received an intraperitoneal injection of 750 μL clodronate (5 mg/mL; Encapsula NanoSciences, CLD8901). This injection was repeated 72 hours later. At this time, the rats were also sensitized with dihydrotachysterol (DHT) at a dose of 10 mg/kg. Twenty-four hours after DHT sensitization, the rats were challenged with a subcutaneous injection of $FeCl_3$. Tissues were collected 96 hours after oral gavage of DHT.

All *in vivo* experiments were conducted using wild-type female Sprague-Dawley rats. However, to investigate the role of immune system, immunodeficient inbred Sprague-Dawley female rats (SRG; Rag2-/- and Il2Rɣ -/-, Charles River, 707) and Rag2KO Sprague-Dawley female rats (Inotiv, SD-$Rag2^{em1sage}$) were used. For the male experiment, Male Sprague-Dawley rats (~130g, Charles River, 400-SAS-SD) were used.

To determine the effect of neutrophil depletion, rats received cyclophosphamide (CPA, MedChem Express, HY17420A) (150mg/kg i.p) 3 days prior to DHT administration, (100mg/k i.p) on the day of DHT administration and a final dose of 50mg/kg i.p was administered 24 hours after $FeCl_3$ injection. In addition, to explore the role of immunosuppressives, Cyclosporine A(CsA, Selleck Chemicals, S2286) was used by starting 7 days prior to DHT administration and continued 24 hour before sacrificing rat. CsA is dissolved in 5% DMSO(sigma, D2650)+40%PEG300(Selleck Chemicals, S6704)+5%Tween80(Selleck Chemicals, S6702)+50%ddH_2O. Vehicle animals only received 5% DMSO+40%PEG300+5%Tween80+50%ddH_2O.

To assess the effect of IL17a blockade, IL17a mAb(BioxCell, BP0173) or IgG(BioxCell, BP0083) was administered i.p. 24 hours prior to DHT administration followed by a repeat dose both i.p. and subcutaneously 24 hours after DHT administration.

To explore the role of MPO inhibitor, rat received 4-aminobenzohydrazide(4-ABAH, Selleck Chemicals, S9874) 24 hours prior to the administration of oral DHT and continued daily till the animals were sacrificed. 4-ABAH is dissolved in 5%

DMSO+40%PEG300+5%Tween80+50%ddH_2O. Vehicle animals only received 5% DMSO+40%PEG300+5%Tween80+50%ddH_2O.

In our study, “n” represents multiple individual animals at a defined time. The different n in various aspects of the study relate to unexpected animal mortality which prevented us from having the same number of animals throughout the study.

RNA extraction

Total RNA was isolated using a hybrid Trizol/RNeasy protocol. Skin samples were collected via 4 mm punch biopsies (Integra Miltex, 3334) and lysed in 600 µL Trizol reagent (Fisher, 15596018) using a handheld homogenizer according to standard protocol. The aqueous phase was separated, mixed with ethanol, and processed using the Qiagen RNeasy Mini Kit (Qiagen, 74134) according to the manufacturer’s instructions.

RNA sequencing

Total RNA was used to generate RNA-seq libraries, which were sequenced on an Illumina HiSeq 3000 platform, Illumina NextSeq 500 and Illumina NovaSeq X Plus. Sequencing reads were aligned to the Rnor_6.0 genome using STAR (version 2.7.1b)[3]. Differential expression analysis was conducted using the DESeq2 package [4]. Counts normalized for sequencing depth were subsequently used for principal component analysis (PCA).

GO analysis and GSEA-KEGG analysis based on differentially expressed genes (DEGs) was conducted using the clusterProfiler R package (v4.1.1)[5]. Gene sets with a P value of less than 0.05 and an FDR value of less than 0.25 were considered statistically significant.

Single cell RNA library construction and sequencing

Single cells were isolated from the dermis of 4 mm dorsal skin samples collected from rats treated with DHT + $FeCl_3$, H_2O, WT, and SRG for 10X Genomics library preparation and sequencing. The procedure was as follows[6]:

Fresh rat skin samples were placed in Dispase II working solution (5 mg/mL) and digested at 37°C for 1 hour. The digested skin was then transferred to a 10 cm petri dish containing pre-chilled HBSS solution to facilitate separation of the dermis from the epidermis. The isolated dermis was subsequently placed in a pre-chilled dermis dissociation buffer containing 100 µg/mL DNase I and 1 mg/mL Collagenase P and incubated at 37°C for 1 hour, with gentle mixing of the tissue suspension every 30 minutes.

The cell suspension was filtered through a 70 μm cell strainer. The filtered suspension was transferred into DMEM/high medium containing 10% FBS and centrifuged at 400 x g, after which the supernatant was discarded. The cell pellet was resuspended in FACS buffer (HBSS with 2% BSA and 0.1 mM EDTA) and filtered again through a 40 μm cell strainer. Following another centrifugation at 400 x g, the pellet was resuspended for cell sorting, yielding approximately 3 x 10^5 live cells for downstream library preparation and sequencing.

Single-cell RNA-seq libraries were prepared using the Chromium Single Cell 3′ Library & Gel Bead Kit v2 (10x Genomics) following the manufacturer's protocol. Sequencing was performed on an Illumina NovaSeq 6000 platform.

Upstream data processing was conducted with CellRanger to generate the expression matrix, followed by cell clustering using Seurat (v4.4) with a minimum threshold of three cells per gene (min.cells = 3) and 200 detected genes per cell (min.features = 200)[7]. Additional filtering was applied based on mitochondrial gene expression, total RNA content, and gene count. Doublets were identified and excluded using the scDblFinder R package (v1.16.0) to ensure that only single cells were retained for downstream analysis [8].

Subsequently, data normalization, standardization, batch correction, and dimensionality reduction were performed to generate the final single-cell gene expression matrix. The processed data were then used for UMAP clustering and cell proportion analysis.

Immunophenotyping with flow cytometry

Skin was harvested for 3 days following subcutaneous challenge. Tissues were washed in PBS, chopped into 1 mm² pieces, and digested in high-glucose DMEM (Gibco, 1195073) containing 1 mg/mL Collagenase P (Sigma, 11213857001) and 100 μg/mL DNase I (Sigma, 10104159001) for 1 hour at 37°C. The tissue suspensions were filtered through a 70 μm cell strainer (Fisher, 22363548), centrifuged at 400 x g for 5 minutes, and resuspended in 2% BSA (Fisher, BP1600100). The suspension was then re-filtered through a 40 μm cell strainer (Fisher, 22363547), centrifuged again, and resuspended.

One million cells suspended in 1 mL of 2% BSA were incubated with antibodies targeting macrophages (CD11b), neutrophils (RP1), and T cells (CD3) for 30 minutes. Following antibody incubation, the cells were resuspended in 2% BSA and analyzed using a BD LSRII flow cytometer. Data were analyzed using FlowJo software.

Histological studies

All tissues were fixed in 10% neutral buffered formalin (Sigma, HT501128) at room temperature overnight. Tissues intended for paraffin sections were transferred to 70% ethanol and submitted to the UCLA Translational Pathology Core Laboratory for paraffin

embedding. Tissues intended for frozen sections were dehydrated in 15% and 30% sucrose solutions, snap-frozen in O.C.T. compound (Fisher, 23730571), and sectioned at 10 μm.

For immunofluorescence staining, tissue sections were post-fixed in 10% neutral buffered formalin for 10 minutes, then simultaneously blocked and permeabilized in a solution containing 10% donkey serum (Sigma, D9663), 1% BSA, and 0.2% Triton X-100 (Sigma, X100) for 1 hour. Sections were incubated with primary antibodies diluted in the serum/BSA/Triton X-100 solution at 4°C overnight. Secondary antibodies were diluted in 10% donkey serum and 1% BSA and incubated with the sections for 1 hour[9].

The sections were then stained with DAPI (Invitrogen, D3571), followed by a 30-minute incubation with OsteoImage Staining Reagent (Lonza, PA1503) for the detection of hydroxyapatite. All sections were mounted with antifade mounting media (Invitrogen, S36936). Images were acquired using a Nikon Eclipse Ti2 confocal microscope (Nikon, USA) and analyzed using NIS-Elements AR software (Nikon, USA) and ImageJ.

Sections for Von Kossa staining (Fisher, NC9239431) were immersed in 5% silver nitrate for 30 minutes under UV light, followed by a 3-minute incubation with 5% sodium thiosulfate. The sections were counterstained with nuclear fast red for 7 minutes.

For Oil Red O staining, sections were first incubated with Mayer's hematoxylin (Sigma, 51275) for 3 minutes, followed by a 1-minute immersion in 60% isopropanol (Fisher, A4164), a 15-minute incubation in 3 mg/mL Oil Red O (Sigma, O1391) diluted in isopropanol, and a final 1-minute immersion in 60% isopropanol.

For Alizarin Red S staining, sections were immersed in 2% Alizarin Red S (Sigma, A5533) for 2 minutes.

Skin calcium quantification

Skin biopsies were cut into small 1mm^2 pieces and incubated overnight in 0.6M HCl at 4°C. Calcium levels in the supernatant were determined via QuantiChrom Calcium assay kit (BioAssay Systems). Calcium quantities were shown normalized to tissue weight.

Alkaline phosphatase activity

Quantification of ALP activity in skin tissue was performed using an alkaline phosphatase activity colorimetric assay kit (Abcam, ab83369). Results were normalized to total protein concentration, which was measured using a BCA assay (Thermo Scientific, 23227).

Antibodies

The following primary antibodies were used for immunostaining, or flow cytometry: For immunostaining, CD45(1:100, Abcam, ab10558);CD68(1:100, Abcam, ab125212); CD31(1:100, Abcam, ab182981); CD3(1:50, BD Biosciences, 554829); CD64(1:100, Thermo Scientific, PA5116539); IL17A(1:50, Affinity Biosciences, DF6127); Myeloperoxidase[EPR20257] (1:100, Abcam, ab208670); Myeloperoxidase(1:50, Novus Biologicals,NBP1-51148); Runx2(1:100, Cell signaling technology, 12556); SOX9(1:100, Thermo Fisher Scientific,14-9765-82); MGP(1:100, Thermo Scientific, PA596323); LYVE1(1:100, Cell signaling technology, 67538). For flow cytometry. CD11b(1:100, BioLegend, 201809), RP1(1:100, BD, 550002), and CD3(1:50, Miltenyi Biotec, 130103127).

Figure S1

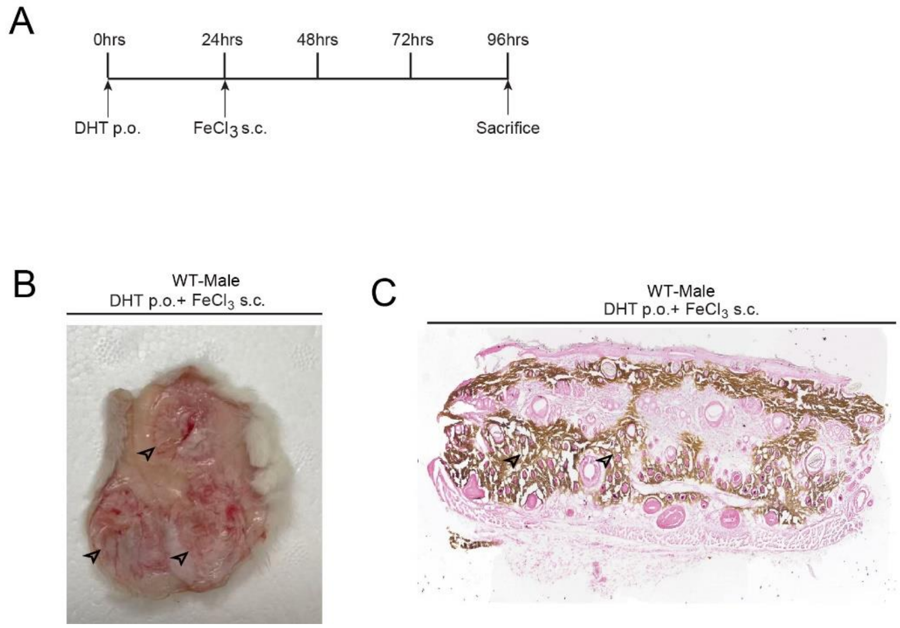

Fig S1. Model of calciphylaxis in male rat.
(A) Experimental scheme in male rodent model of calciphylaxis with similar administration of DHT orally followed by subcutaneous injection of $FeCl_3$ as in female animals. **(B)** Gross images of rat dermal tissue in male rats demonstrating subcutaneous calcific nodules (arrows). **(C)** Representative Von Kossa-stained images of rat dermal tissue highlighting calcified regions (arrows) (n=5 animals).

Figure S2

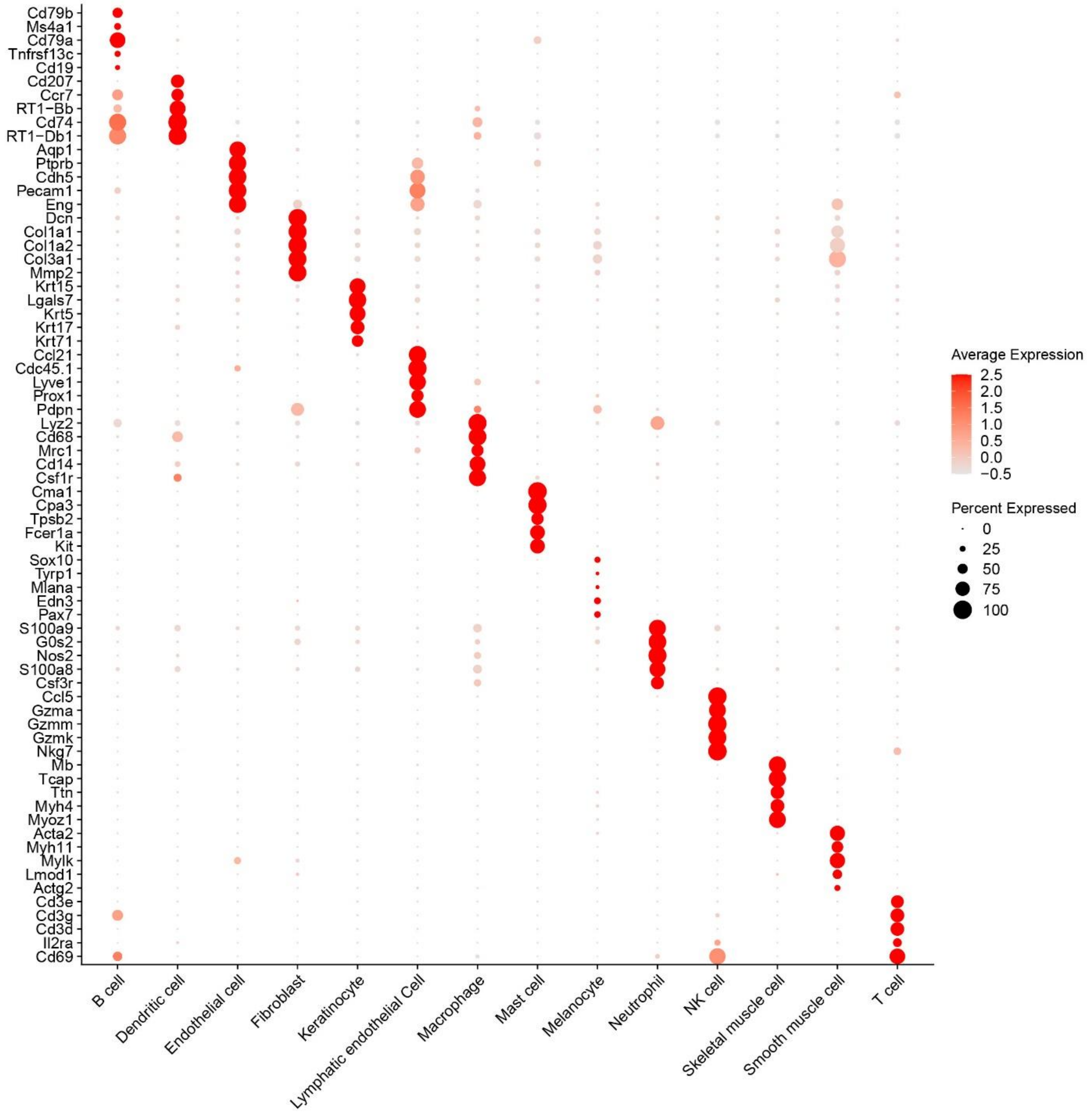

Fig S2. Single-cell RNA sequencing of skin of DHT p.o.+$FeCl_3$ s.c. and vehicle p.o. +H_2O s.c. treated animals. Canonical genes used to characterize various cell population in the skin.

Figure S3

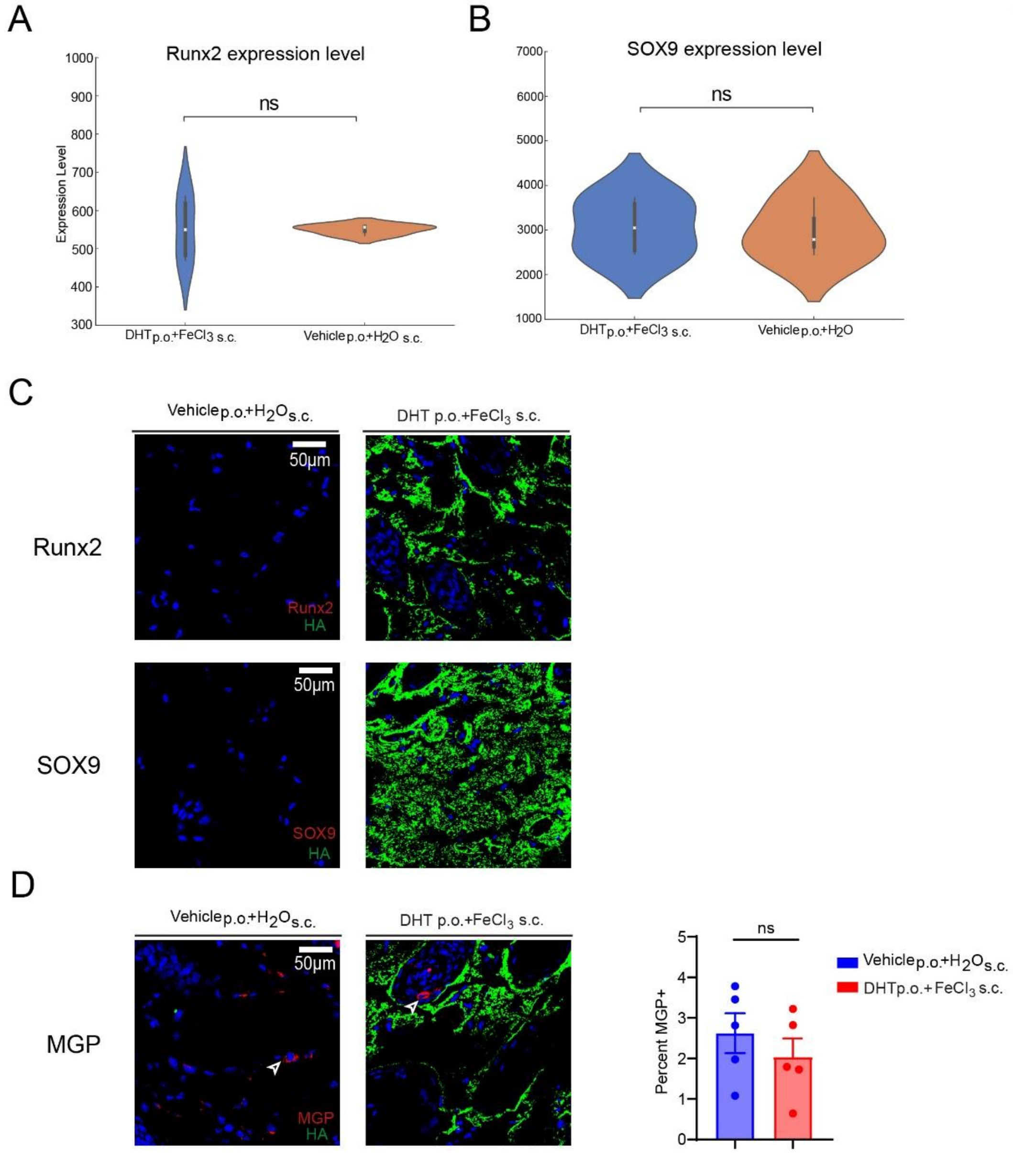

Fig S3. Runx2, SOX9 and MGP expression is not markedly altered in DHT+$FeCl_3$-treated tissue. (A–B) Violin plots showing expression levels of (A) Runx2 and (B) SOX9 from bulk RNA-seq data comparing DHT p.o. +$FeCl_3$ s.c. group (n=4 animals) and vehicle p.o. +H_2O s.c. control (n=3 animals). No significant difference was observed (ns, not significant). **(C)** Representative immunofluorescence images of skin sections stained for Runx2 and SOX9. Red: Runx2, SOX9; Green: HA; Blue: nuclei (DAPI). **(D)** Representative immunofluorescence images and quantification of MGP in the rat skin. Red: MGP; Green: HA; Blue: nuclei (DAPI). (n=5 animals/per group). Data are represented as mean ± SEM. ns: no statistic difference, 2-tailed Student's t test.

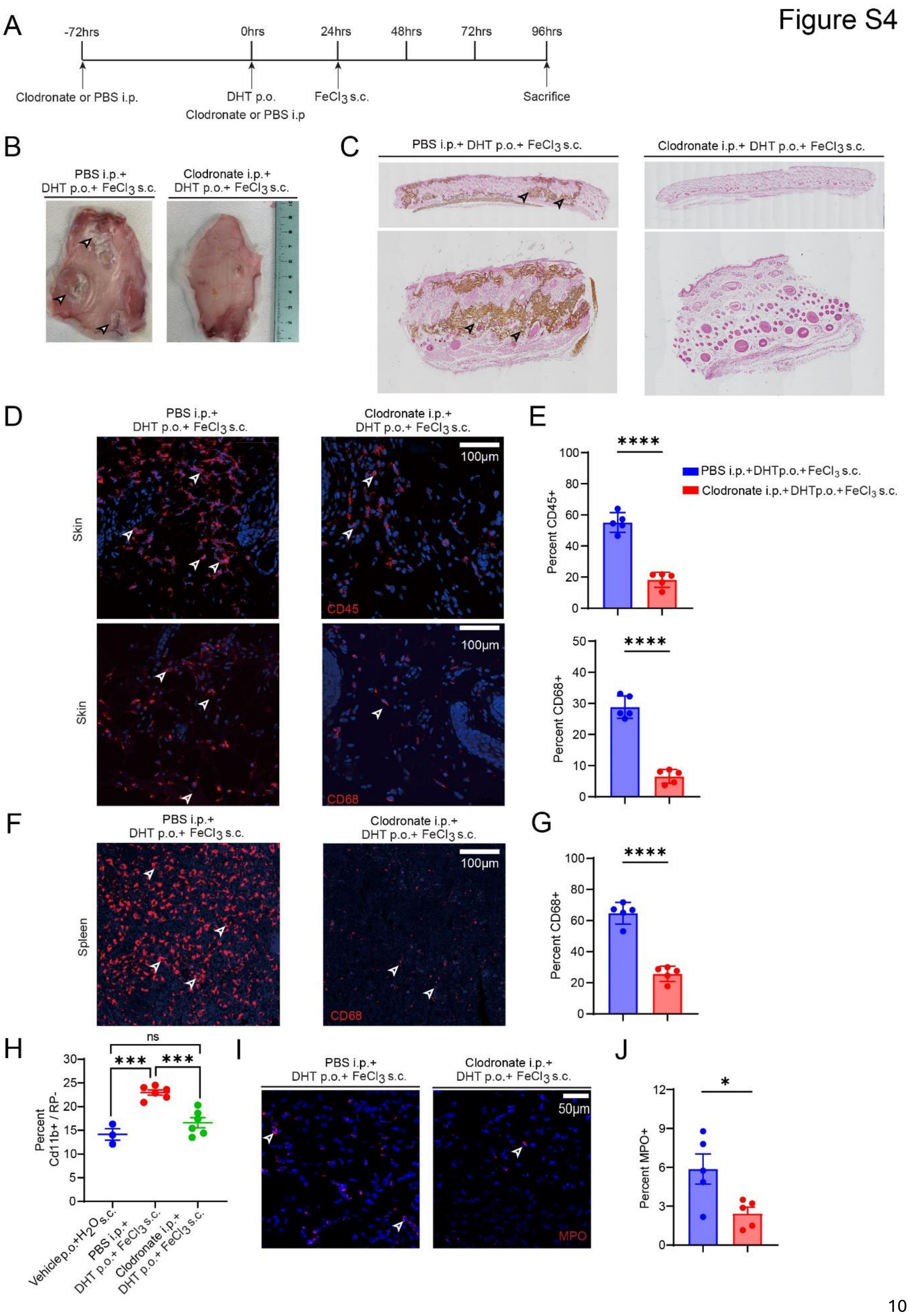

Figure S4
A
-72hrs
0hrs
24hrs
48hrs
72hrs
96hrs
Clodronate or PBS i.p.
DHT p.o.
Clodronate or PBS i.p
FeCl3 s.c.
Sacrifice
B
PBS i.p.+ DHT p.o.+ FeCl3 s.c.
Clodronate i.p.+ DHT p.o.+ FeCl3 s.c.
C
PBS i.p.+ DHT p.o.+ FeCl3 s.c.
Clodronate i.p.+ DHT p.o.+ FeCl3 s.c.
D
PBS i.p.+ DHT p.o.+ FeCl3 s.c.
Clodronate i.p.+ DHT p.o.+ FeCl3 s.c.
Skin
100µm
CD45
CD68
E
Percent CD45+
Percent CD68+
****
PBS i.p.+DHTp.o.+FeCl3 s.c.
Clodronate i.p.+DHTp.o.+FeCl3 s.c.
F
Spleen
G
Percent CD68+
H
Percent Cd11b+ / RP-
ns
***
Vehiclep.o+H2O s.c.
PBS i.p.+ DHT p.o.+ FeCl3 s.c.
Clodronate i.p.+ DHT p.o.+ FeCl3 s.c.
I
50µm
MPO
J
Percent MPO+
*

Fig S4. Ablation of macrophages with the drug clodronate prevents ectopic calcification.

(**A**) Experimental scheme for administration of clodronate in rodent models of calciphylaxis. (**B**) Gross images of rat dermal tissue following treatment with clodronate or PBS. Black arrows show visible regions of calcification. (**C**) Representative Von Kossa-stained images of rat dermal tissue highlighting calcified regions (arrows). (**D**) Immunofluorescent staining for CD45 and CD68 in rat dermal tissue treated with clodronate or PBS and **(E)** quantification of macrophages/CD45 cells (Representative images, n=5 animals/per group). **(F)** Immunofluorescent analysis of CD68+ cells in rat splenic tissue following treatment with clodronate or PBS and **(G)** quantification (n=5 animals/per group)**. (H)** Representative flow cytometry analysis for circulating bone marrow monocytes of rats treated with vehicle p.o. + H_2O s.c. and DHT p.o.+$FeCl_3$ s.c. after clodronate or PBS administration (H_2O n=3 animals, clodronate n=6 animals, PBS n=6 animals). **(I)** Immunofluorescence staining pictures of MPO in skin tissue and **(J)** quantification of MPO^+ cells (Representative images, n=5 animals/per group). Data are represented as mean ± SEM. ****$P < 0.0001$; ***$P < 0.001$; *$P < 0.05$, 2-tailed Student's t test.

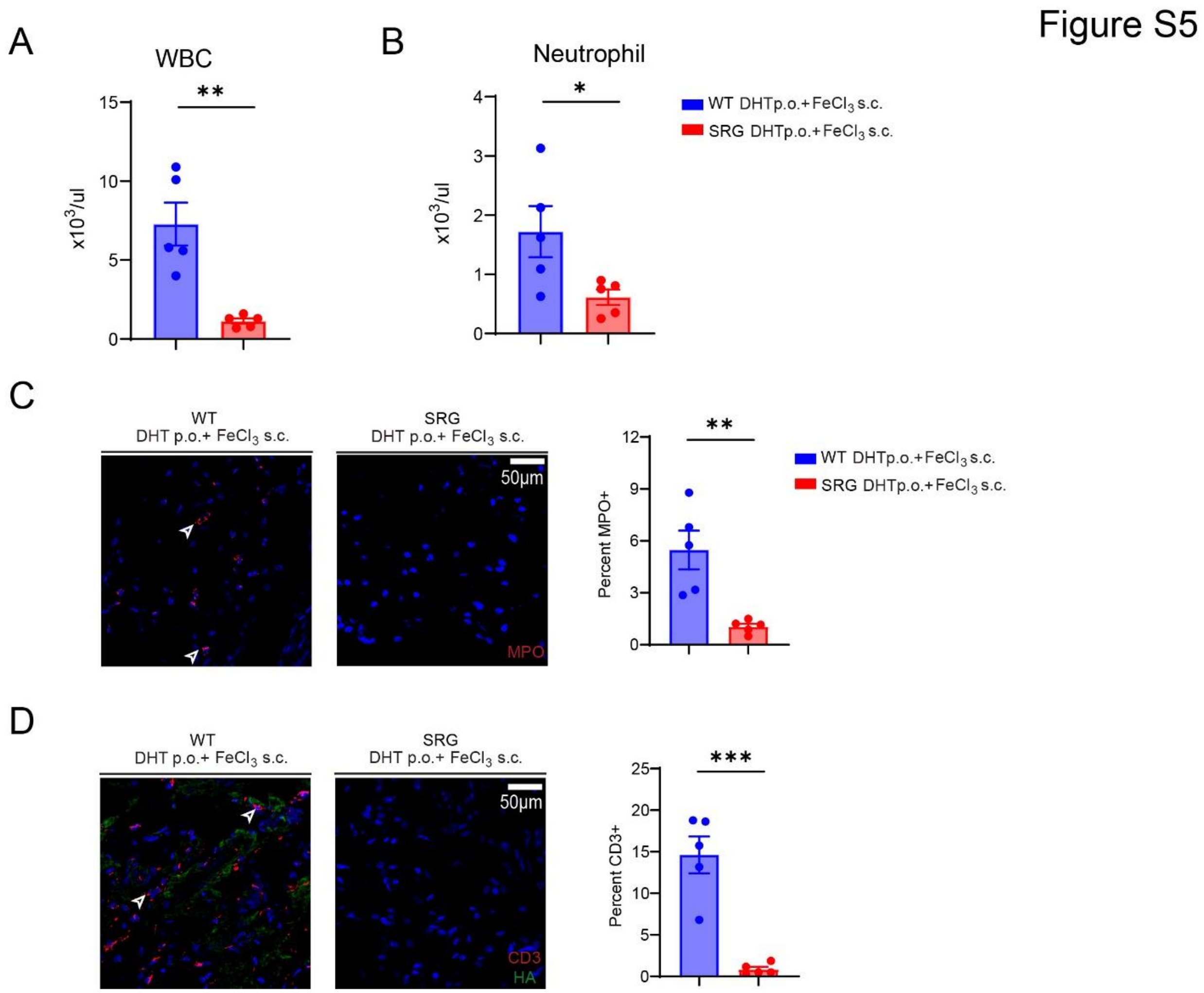

Fig S5. Reduced circulating white blood cells and neutrophils and decreased local immune infiltration in SRG rats.
(A) Quantification of total white blood cells and **(B)** circulating neutrophils in WT and SRG rats following DHT oral administration and subcutaneous $FeCl_3$ injection (n=5 animals/per group). **(C)** Immunofluorescence staining and quantification of MPO^+ cells and **(D)** $CD3^+$ T cells in WT vs. SRG rats skin tissue (Representative images, n=5 animals/per group)**.** Data are represented as mean ± SEM. ***$P < 0.001$; **$P < 0.01$; *$P < 0.05$, 2-tailed Student's t test.

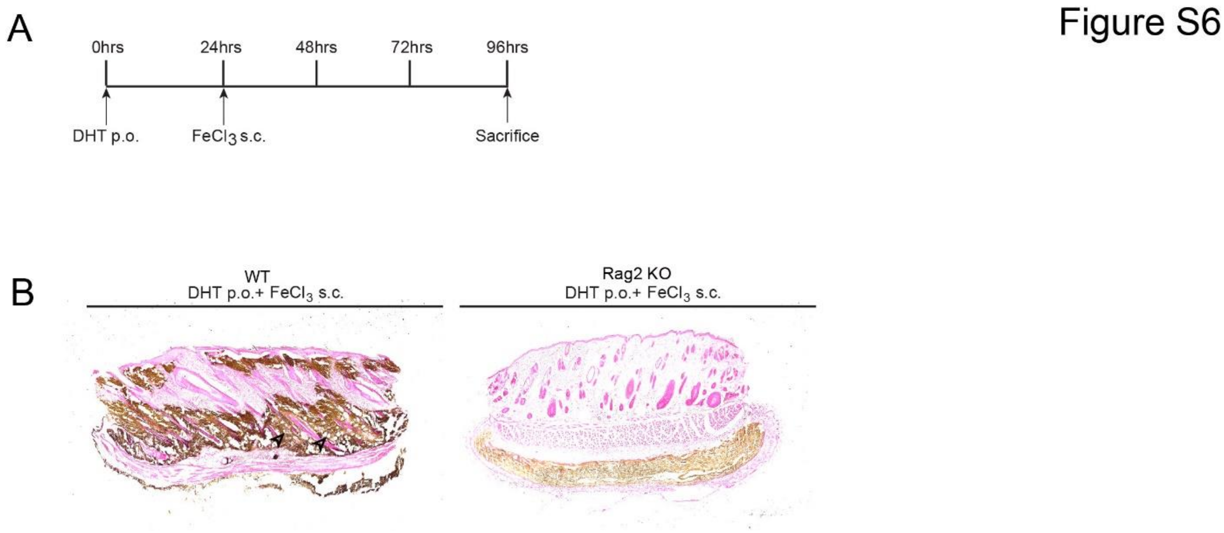

Fig S6. Rag2KO rats are resistant to the development of ectopic calcification.

(**A**) Experimental strategy with WT/ Rag2KO rats administered DHT + $FeCl_3$. (**B**) Representative Von Kossa-stained image of rat dermal tissue highlighting calcification in WT but not in Rag2KO animal skin (n=3 animals/per group).

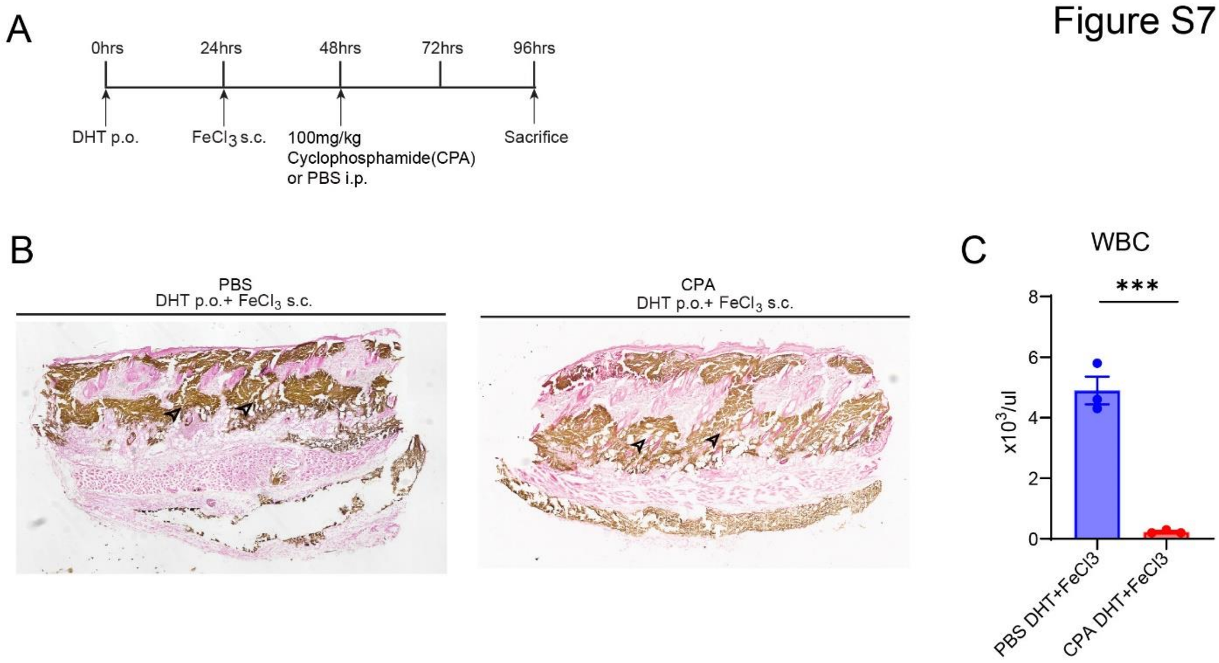

Fig S7 Cyclophosphamide treatment after $FeCl_3$ administration reduces peripheral leukocyte count but does not alter tissue calcification.
(A) Experimental strategy showing DHT and $FeCl_3$ administration followed by intraperitoneal injection of cyclophosphamide (CPA, 100 mg/kg) or PBS at 48 hours.
(B) Representative Von Kossa-stained skin sections from CPA- and PBS-treated groups showing comparable calcified areas (black arrow show calcific deposits, n=3 animals/per group).
(C) Quantification of peripheral white blood cell (WBC) counts demonstrates dramatic leukopenia in CPA-treated rats. Data are represented as mean ± SEM.***$P < 0.001$, 2-tailed Student's t test.

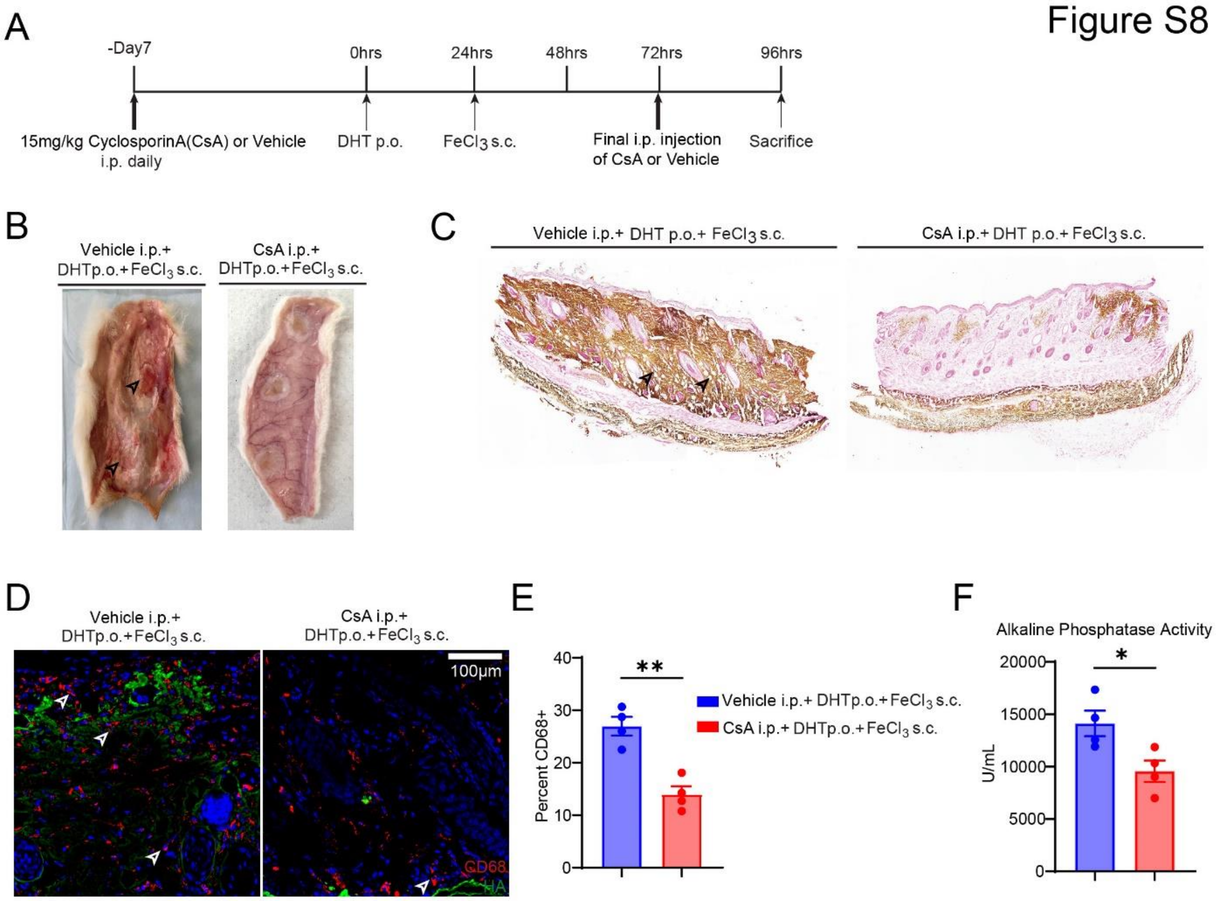

Fig S8. T cell inhibitor, cyclosporine blocks the formation of ectopic calcification.

(**A**) Experimental strategy for administration of T cell inhibitor cyclosporine (Cyclosporin A) in WT rats administered with DHT p.o.+$FeCl_3$ s.c.. (**B**) Gross images of rat dermal tissue in Vehicle and Cyclosporin A treated rats. Black arrows show visible regions where calcification is present. (**C**) Representative Von Kossa-stained image of rat dermal tissue highlighting calcification after DHT p.o.+$FeCl_3$ s.c. (n=4 animals/per group). (**D-E**) Immunofluorescent staining of CD68-positive cells in Vehicle and CyclosporinA treated rats (Representative images, n=4 animals/per group). **(F)** Assessment of alkaline phosphatase activity in rat dermal tissues treated with vehicle or cyclosporin A (n=4 animals/per group). Data are represented as mean ± SEM. $^{**}P < 0.01$; $^{*}P < 0.05$, 2-tailed Student's t test.

Figure S9

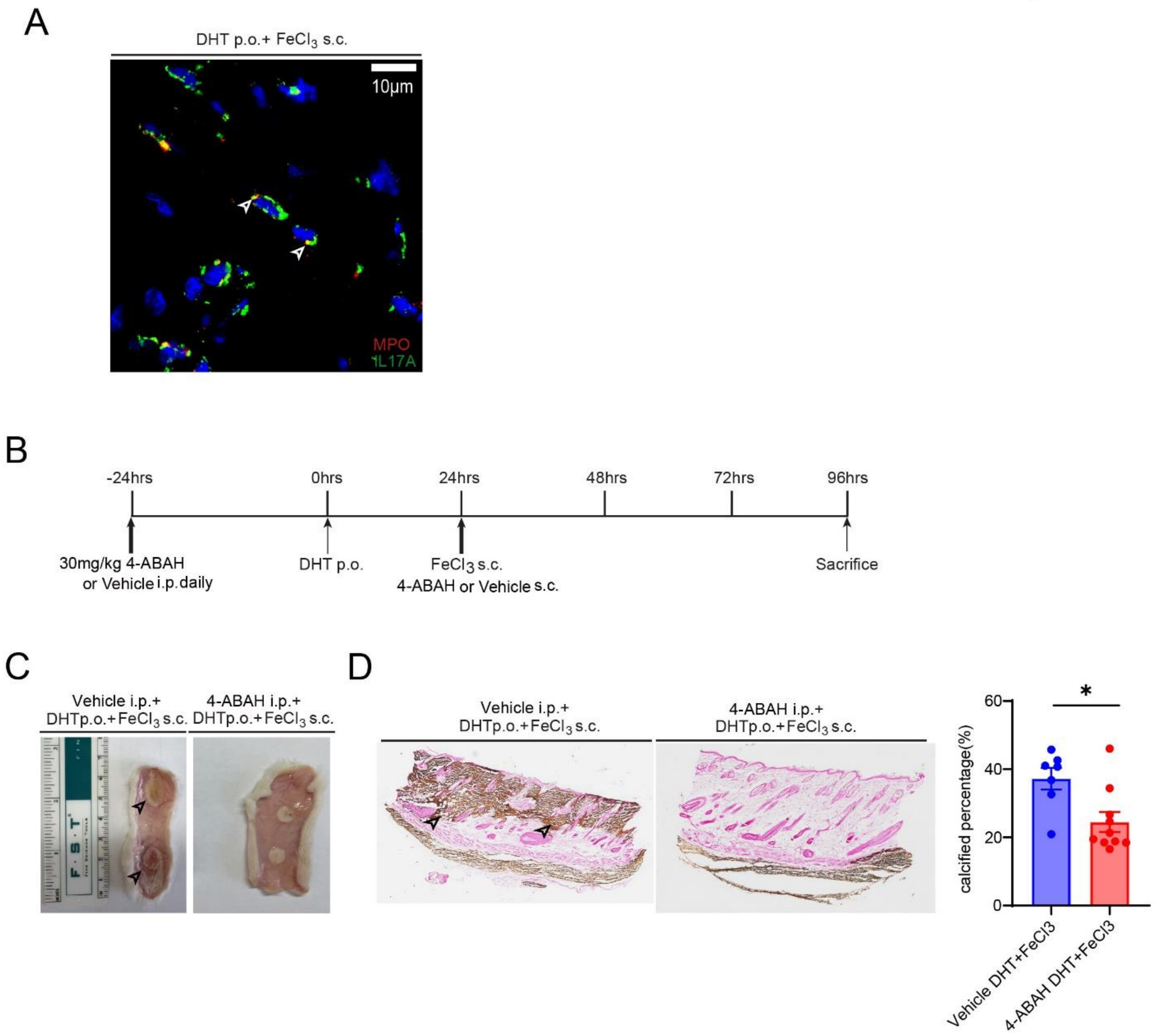

Fig S9. Inhibition of MPO with 4-ABAH(4-Aminobenzohydrazide) attenuates ectopic calcification in a rodent model of calciphylaxis.

(A) Representative image showing colocalization of IL-17A (green) and MPO (red) in DHT +$FeCl_3$ treated skin. **(B)** Schematic outlining the experimental strategy used in the study with MPO inhibition started 24 hours prior to DHT administration and continued for the rest of the study, followed by a single subcutaneous dose at 24 hours alongside $FeCl_3$. **(C)** Macroscopic section of dorsal skin from rats, with black arrows indicating visibly calcified regions (arrows) in control but not 4-ABAH groups. **(D)** Representative images of skin sections stained with Von Kossa to visualize calcium deposition (indicated by arrows). Quantification shows a robust reduction in the percentage of calcified tissue in vehicle-(n=7 animals) and 4-ABAH-(n=10 animals) treated rats administered with DHT p.o.+$FeCl_3$ s.c.. Data are represented as mean ± SEM. *$P < 0.05$, 2-tailed Student's t test.

Table S1. Serum biochemistry 24 hours after DHT or H_2O administration.

	H_2O(n=5)	DHT 24h (n=3)
Calcium (mg/dl)	13.00±0.6218	14.67±0.4163*
Phosphorus (mg/dl)	11.91±0.9607	14.43±0.6028**
Creatinine (mg/dl)	0.28±0.05	0.23±0.0577
BUN (mg/dl)	12.00±1.7321	18.00±8.7178

Table S1. Serum biochemistry 24 hours after DHT or H_2O administration.

Serum chemistry in rats 24 hours following treatment with DHT or water (n=3 animals). There was a substantial increase in serum calcium (*$P < 0.05$) and phosphorus (**$P < 0.01$) in the animals receiving DHT compared to those that received H_2O group (n=5 animals). Creatinine and BUN levels showed no substantial changes between groups.

Table S2. WBC counts in rats treated with Clodronate and PBS following $DHT+FeCl_3$.

	PBS<br>$DHT+FeCl_3$(n=5)	Clodronate<br>$DHT+FeCl_3$(n=5)
WBC (10^3/ul)	6.32±1.1692	6.6±2.9521
Neutrophil (10^3/ul)	1.36±0.5849	1.29±0.4658

Table S2. WBC counts in rats treated with Clodronate and PBS following $DHT+FeCl_3$.

Peripheral blood WBC or neutrophil counts between the clodronate- and PBS-treated groups (n=5 animals/per group, $P > 0.05$).

Table S3. Serum biochemistry in clodronate- and PBS-treated rats 72 hours after $DHT+FeCl_3$.

	PBS $DHT+FeCl_3$(n=4)	Clodronate $DHT+FeCl_3$(n=4)
Calcium (mg/dl)	14.00±1.5937	12.5±0.8602
Phosphorus (mg/dl)	12.93±1.12	10.68±0.6021*
Creatinine (mg/dl)	0.42±0.1258	0.30±0.0816
BUN (mg/dl)	68.25±27.9091	23.25±11.9269

Table S3. Serum biochemistry in clodronate- and PBS-treated rats 72 hours after $DHT+FeCl_3$.

Serum chemistry in rats treated with clodronate (n=4 animals) or PBS following $DHT+FeCl_3$ administration. Decreased serum phosphorus levels (*$P < 0.05$) compared to PBS-treated controls (n=4 animals) was noted. Calcium, creatinine, and BUN levels were also lower in the clodronate group, but no statistical significance was observed.

Table S4. Serum biochemistry in WT and SRG rats 72 hours after DHT+$FeCl_3$.

	WT<br>DHT+$FeCl_3$(n=4)	SRG<br>DHT+$FeCl_3$(n=5)
Calcium (mg/dl)	14.60±1.2987	12.72±0.5891*
Phosphorus (mg/dl)	12.77±2.4391	11.42±1.8579
Creatinine (mg/dl)	0.47±0.2217	0.48±0.1095
BUN (mg/dl)	58.25±45.2645	44.80±24.5906

Table S4. Serum biochemistry in WT and SRG rats 72 hours after DHT+$FeCl_3$.
Serum chemistry in SRG or WT rats (n=5 animals) following DHT+$FeCl_3$ (*$P < 0.05$) compared to WT (n=4 animals). Phosphorus, creatinine, and BUN levels were also lower in SRG rats, but the differences were not statistically significant.

Table S5. Serum biochemistry in rats treated with CPA (cyclophosphamide) or PBS at 72 hours following DHT+$FeCl_3$ administration.

	PBS<br>DHT+$FeCl_3$(n=4)	CPA<br>DHT+$FeCl_3$(n=5)
Calcium (mg/dl)	13.83±0.9215	12.85±2.5093
Phosphorus (mg/dl)	13.85±2.0634	11.77±2.7109
Creatinine (mg/dl)	0.65±0.3317	0.75±0.4933
BUN (mg/dl)	73.5±44.1399	96.5±47.7947

Table S5. Serum biochemistry in rats treated with CPA (cyclophosphamide) or PBS at 72 hours following DHT+$FeCl_3$ administration.

Serum biochemistry in CPA-treated rats (n=5 animals) compared to PBS treated controls (n=4 animals) ($P > 0.05$)

Table S6. Serum biochemistry in animals that received IL-17A mAb or IgG 72 hours after DHT + $FeCl_3$.

	IgG DHT+$FeCl_3$(n=5)	IL17mAb DHT+$FeCl_3$(n=6)
Calcium (mg/dl)	13.52±1.6941	12.07±2.419
Phosphorus (mg/dl)	13.4±1.6378	10.95±0.9138**
Creatinine (mg/dl)	0.4±0.1250	0.25±0.0836*
BUN (mg/dl)	67.00±35.6791	21.33±5.4650**

Table S6. Serum biochemistry in animals that received IL-17A mAb or IgG 72 hours after DHT + $FeCl_3$.

Serum chemistry in animals treated with IL-17A monoclonal antibody (n=6 animals) or IgG demonstrating markedly reduced serum phosphorus (**$P < 0.01$), creatinine (*$P < 0.05$), and BUN levels (**$P < 0.01$) compared to IgG-treated controls (n=5 animals). Calcium levels also trended lower but without statistical significance.

Table S7. Serum biochemistry at 72 hours post–DHT + $FeCl_3$ in rats with the MPO inhibitor 4-ABAH or vehicle.

	Vehicle DHT+$FeCl_3$(n=5)	4-ABAH DHT+$FeCl_3$(n=5)
Calcium (mg/dl)	13.26±2.3977	12.86±2.2468
Phosphorus (mg/dl)	12.58±2.7362	10.76±1.9139
Creatinine (mg/dl)	0.52±0.3697	0.36±0.05472
BUN (mg/dl)	59.60±38.9572	31.2±17.8805

Table S7. Serum biochemistry at 72 hours post–DHT + $FeCl_3$ in rats with the MPO inhibitor 4-ABAH or vehicle.

Serum biochemistry in animals treated with 4-ABAH (n=5 animals) or Vehicle-treated controls (n=5 animals). MPO treated animals exhibited lower levels of serum calcium, phosphorus, creatinine, and BUN, but these differences were not statistically significant.

References

1. SELYE H, et al. Cutaneous molt induced by calciphylaxis in the rat. Science. 1961. 134(3493):1876-7.
2. Miller S, et al. Cutaneous calciphylactic reactions in the mouse and the rat and the effects of diphosphonates on the reaction in the rat. J Pathol. 1984.142(1):7-13.
3. Dobin A, et al. STAR: ultrafast universal RNA-seq aligner. Bioinformatics, 2013. 29(1): p. 15-21.
4. Love M.I., et al. Moderated estimation of fold change and dispersion for RNA-seq data with DESeq2. Genome Biol, 2014. 15(12): p. 550.
5. Wu T, et al. clusterProfiler 4.0: A universal enrichment tool for interpreting omics data. Innovation (Camb). 2021.2(3):100141.
6. Lou F, et al. Protocol for Flow Cytometric Detection of Immune Cell Infiltration in the Epidermis and Dermis of a Psoriasis Mouse Model. STAR Protoc. 2020. 1(3):100115.
7. Stuart T, et al. Comprehensive Integration of Single-Cell Data. Cell, 2019. 177(7): p. 1888-1902 e21.
8. Germain PL, et al. Doublet identification in single-cell sequencing data using *scDblFinder*. F1000Res. 2021. 10:979.
9. Li S, et al. Cardiomyocytes disrupt pyrimidine biosynthesis in nonmyocytes to regulate heart repair. J Clin Invest. 2022. 132(2):e149711.
